# Maximizing the ovarian reserve in mice by evading LINE-1 genotoxicity

**DOI:** 10.1038/s41467-019-14055-8

**Published:** 2020-01-16

**Authors:** Marla E. Tharp, Safia Malki, Alex Bortvin

**Affiliations:** 1 Department of Embryology, Carnegie Institution for Science, Baltimore, MD 21218 USA; 2 Department of Biology, Johns Hopkins University, Baltimore, MD 21218 USA

**Keywords:** Oogenesis, Transposition

## Abstract

Female reproductive success critically depends on the size and quality of a finite ovarian reserve. Paradoxically, mammals eliminate up to 80% of the initial oocyte pool through the enigmatic process of fetal oocyte attrition (FOA). Here, we interrogate the striking correlation of FOA with retrotransposon LINE-1 (L1) expression in mice to understand how L1 activity influences FOA and its biological relevance. We report that L1 activity triggers FOA through DNA damage-driven apoptosis and the complement system of immunity. We demonstrate this by combined inhibition of L1 reverse transcriptase activity and the Chk2-dependent DNA damage checkpoint to prevent FOA. Remarkably, reverse transcriptase inhibitor AZT-treated Chk2 mutant oocytes that evade FOA initially accumulate, but subsequently resolve, L1-instigated genotoxic threats independent of piRNAs and differentiate, resulting in an increased functional ovarian reserve. We conclude that FOA serves as quality control for oocyte genome integrity, and is not obligatory for oogenesis nor fertility.

## Abstract (web summary)

Mammals lose up to 80% of their finite oocyte supply during fetal development. Here the authors interrogate mechanisms of fetal oocyte attrition in mice, driven by the simultaneous upregulation of LINE-1 retrotransposon activity and inhibit these mechanisms to increase the functional ovarian reserve.

## Introduction

Oogenesis programs across metazoans reflect diverse reproductive strategies observed in nature. Species that succeed by producing large numbers of offspring, like *Drosophila melanogaster* and *Caenorhabditis elegans*, rely on robust germline stem cell-based oogenesis programs to ensure the egg supply throughout reproductive life^1^. In contrast, mammalian females are endowed with a non-renewable ovarian reserve to ultimately produce few offspring^2–4^. Thus, female fertility and reproductive lifespan in mammals critically depend on the size and quality of the ovarian reserve of primordial follicles, a supply of arrested oocytes and associated somatic cells established by birth^5–8^. Paradoxically, the ovarian reserve of primordial follicles at birth reflects only a smaller share (~20% in humans) of all oocytes initially specified in the fetal ovary^9,10^. The majority of fetal oocytes generated are lost by fetal oocyte attrition (FOA), a conserved phenomenon among mammals^9,11–14^. Since the discovery of FOA in the 1960s, however, the mechanisms and underlying developmental or physiological rationale of this process remain debated. Over the ensuing decades, inadequate oocyte support, oocyte self-sacrifice, and meiotic DNA damage featured prominently in models attempting to explain this phenomenon^15–17^. However, the biological significance of FOA is still emerging, and it remains unknown whether preventing FOA, if possible at all, would benefit or perturb fertility.

**Fig. 1:**
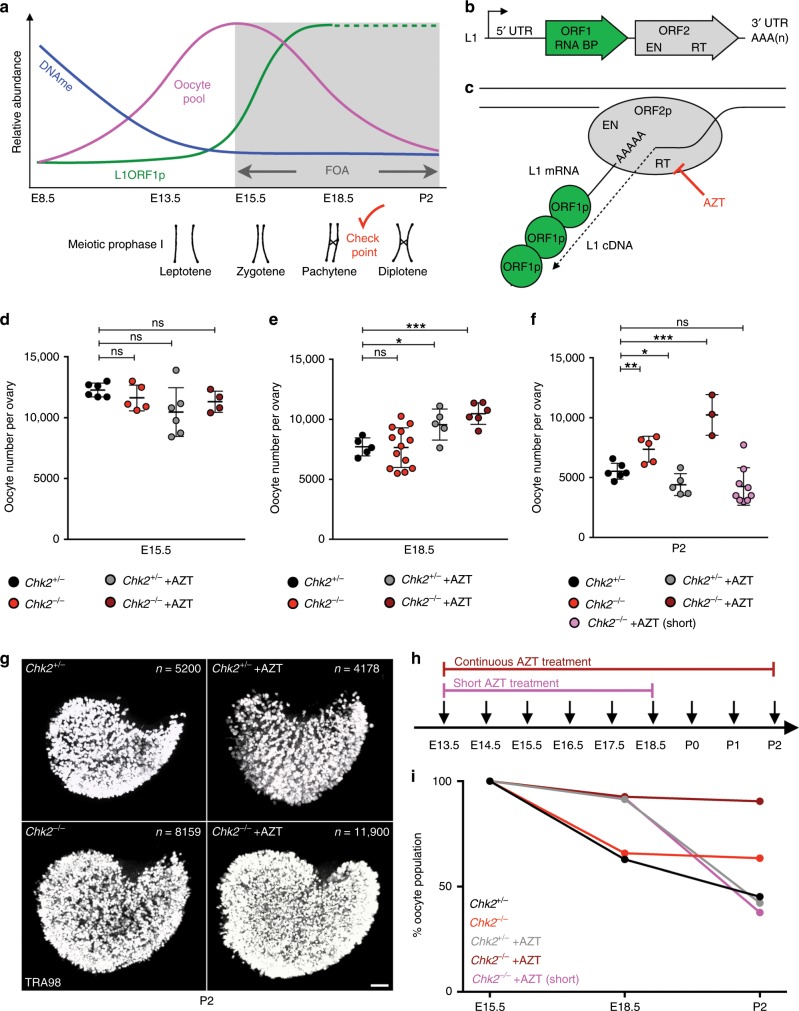
Experimental evasion of L1-instigated FOA. **a** Model of developmental programs influencing the oocyte pool (pink) and FOA (gray). DNA methylation (DNAme) is erased genome-wide for epigenetic reprogramming (blue). Loss of DNAme on L1 sequences permits L1 ORF1p expression at E15.5 that peaks around E18.5 (green). How L1 expression is regulated beyond E18.5 is unknown (dotted green). Entry into meiotic prophase I (consisting of leptotene, zygotene, pachytene, and diplotene substages) involves complex chromosome rearrangements and DNA breakage for meiotic recombination. Red checkmark represents DNA damage checkpoint activation after meiotic recombination is completed. **b** Model of L1 element structure. L1 encodes two proteins, ORF1p that binds RNA (RNA BP) and ORF2p that possesses the catalytic activities for retrotransposition. These are endonuclease (EN) and reverse transcriptase (RT) activities. The RT activity of ORF2p can be inhibited using the drug AZT (red). **c** Model of L1 reverse transcription mechanism. L1 ribonucleoprotein complexes (RNPs) contain L1 ORF1p associated with L1 RNA and L1 ORF2p in addition to other factors. L1 RNPs associate with genomic DNA where EN activity of ORF2p cuts DNA and reverse transcriptase activity of ORF2p generates L1 cDNA using the L1 RNA template. **d**, **e** Oocyte number per ovary at **d** E15.5 and **e** E18.5. **f** Oocyte number per ovary at P2 in *Chk2*^*+/−*^ and *Chk2*^*−/−*^ untreated and AZT-treated ovaries. For P2 ovaries, AZT was administered either daily from E13.5 until P2 (default), or daily from E13.5 until E18.5 (short). **d**–**f** Dots indicate independent ovary samples; data are mean + SD; *n* > 3 ovaries per sample from at least three different embryos and two different litters. See Supplementary Data 1a. Stats by two-tailed unpaired Student’s *t*-test, ns *p* > 0.05; *p < 0.05; **p < 0.01; ***p < 0.001. See Supplementary Data 1b. **g** TRA98 labeling of representative P2 *Chk2*^*+/*−^ and *Chk2*^−*/*−^ untreated and AZT-treated ovaries. *n* oocytes per ovary shown. Scale bar:100 μm. **h** Schematic for continuous and short AZT administration regimens. **i** Dynamics of FOA between E15.5 and P2 visualized as percent of maximum starting oocyte number at E15.5. *Chk2*^*+/*−^ and *Chk2*^*−/*−^ untreated and AZT-treated ovaries (default continuous and short AZT treatments) are shown.

In addition to the above-mentioned models of FOA, we have recently implicated a non-LTR retrotransposon LINE-1 (L1) in FOA in mice^18^ (Fig. 1a). L1 elements are active and highly abundant, with full-length and truncated L1 elements accounting for about 20% the human and mouse genomes^19–21^. A generic full-length L1 element is 7 kb long with two open-reading frames, *ORF1* and *ORF2* (Fig. 1b)^22–24^. *ORF1* encodes an RNA-binding protein that functions as a major component of the L1 ribonucleoprotein particle (RNP) and a nucleic acid chaperone during L1 insertion^25,26^. ORF2p has endonuclease and reverse transcriptase activities that are crucial for L1 retrotransposition^27,28^. A generalized mechanism of L1 retrotransposition involves DNA nicking by ORF2p, annealing of the L1 poly(A)-tail to the released DNA strand, which also provides a 3′-OH end to prime L1 reverse transcription, second strand synthesis, and ligation of the final product to genomic DNA^27,29^ (Fig. 1c).

Prior studies showed that L1 overexpression in mammalian cultured cells causes accumulation of DNA damage, activation of respective checkpoints, and cell death^28,30^. Interestingly, both reverse transcriptase and endonuclease activities of ORF2p individually are detrimental to cells^30^. To protect genomes from the harmful L1 activity, multiple epigenetic and RNA-based mechanisms of transposable element control have evolved^24,31,32^. These mechanisms are especially critical for germ cells, not only for viability but also to prevent the transmission of mutations caused by L1 mobilization to subsequent generations. Despite this, epigenetic remodeling of primordial germ cells necessitates the genome-wide removal of a primary L1 defense mechanism in the form of repressive DNA methylation, creating permissive conditions for L1 expression^18,33–35^. Male germ cells use Piwi-interacting (pi)RNAs, DNA methylation, and histone modifications to rapidly repress L1^36–39^. In contrast, oocytes re-acquire DNA methylation only during postnatal differentiation and, therefore, remain undermethylated and express L1 during meiotic prophase I^18,34,35^.

In our prior work, we reported a striking correlation of L1 expression with DNA damage and fate of fetal oocytes (Fig. 1a)^18^. We corroborated the important role of L1 in FOA in experiments using azidothymidine (AZT), an inhibitor of reverse transcriptase including that of L1 ORF2p (Fig. 1c)^40,41^. Interestingly, the protective effect of AZT on fetal oocytes is only temporary, limited to the early substages of meiotic prophase. This observation suggested the involvement of an additional mechanism(s) in FOA. We hypothesized that these mechanisms are related to DNA damage due to persisting endonuclease activity of L1 upon AZT treatment and a DNA damage checkpoint employed during meiotic prophase I^42,43^.

In this work, we extended the analysis of L1 involvement in FOA to *Chk2-*mutant mice lacking the critical DNA damage checkpoint protein in meiotic prophase I oocytes^43,44^. We report that the combined inhibition of L1 reverse transcriptase and abrogation of the DNA damage checkpoint in mice result in maximized ovarian reserve. Interestingly, despite the initial L1 overexpression and DNA damage, oocytes evading FOA are capable of reducing this genotoxic stress. Furthermore, we observed no reduction of fertility in animals with increased ovarian reserve. These observations show that contrary to some existing models, FOA is not an essential developmental program but an oocyte quality control that eliminates poor quality oocytes from entering the ovarian reserve.

## Results

### DNA damage checkpoint activity in FOA

To test if FOA is triggered by both L1 reverse transcriptase activity and L1-instigated activation of the DNA damage checkpoint, we examined the role of checkpoint kinase 2 (CHK2). CHK2 transduces information regarding unrepaired DNA double-stranded breaks (DSBs) and other common meiotic defects, such as asynapsis to apoptotic machinery in the mid-pachytene stage of meiotic prophase I ^43^. *Chk2*^−/−^ mice are viable, fertile and, unlike in wild-type mice, unrepaired DSBs do not cause oocyte death^43^. However, whether CHK2 plays a role in FOA under physiological conditions is unknown.

Since CHK2 activity is related to cell cycle control, we first assessed whether loss of CHK2 influences oocyte proliferation or meiotic progression. We compared oocyte numbers between *Chk2*^*−/−*^ mutant and *Chk2*^*+/*−^ control ovaries at E15.5, the stage at which the oocyte supply reaches its maximum size and the onset of FOA. At this timepoint, we observed no difference in oocyte numbers between *Chk2*^−*/−*^ and *Chk2*^*+/−*^ ovaries, suggesting that CHK2 does not impact germ cell proliferation prior to meiotic entry (Fig. 1d and Supplementary Fig. 1a–c and Supplementary Data 1a–d). We next analyzed the role of CHK2 in meiotic prophase I progression by quantifying the percentage of oocytes at preleptotene, leptotene, zygotene, pachytene, and diplotene stages based on morphology of the synaptonemal complex (Supplementary Fig. 2a). We found no difference in meiotic progression between *Chk2*^*−/−*^ and *Chk2*^*+/−*^ oocytes at E15.5 (Supplementary Fig. 2b). However, at E18.5, we did observe an over-representation of diplotene stage oocytes likely spared in the absence of DNA damage checkpoint activation in the mid-pachytene stage (Supplementary Fig. 2c and Supplementary Data 2a, b).

To determine the role for CHK2 in FOA, we compared oocyte numbers between *Chk2*^*−/−*^ and *Chk2*^*+/*−^ ovaries at E18.5, when nearly half of the oocyte supply is eliminated in wild-type mice, and at postnatal day 2 (P2), after an additional smaller population of oocytes is eliminated and the endpoint of FOA^18^. At E18.5, and all other stages prior to DNA damage checkpoint activation, the oocyte number is comparable between *Chk2*^*−/−*^ and *Chk2*^*+/−*^ ovaries (Fig. 1e and Supplementary Fig. 3a). However, by P2, *Chk2*^*−/−*^ ovaries contained significantly more oocytes than P2 *Chk2*^*+/−*^ ovaries, and a comparable number to E18.5 *Chk2*^*−/−*^ ovaries (Fig. 1f, g). These data suggest a role for CHK2 in FOA beginning at E18.5, consistent with timing of DNA damage checkpoint activation in the mid-pachytene stage, and supported by upregulation of the CHK2 target TAp63 at E18.5, but not at E15.5 when the dominant negative isoform is expressed (Supplementary Fig. 4a, b).

Since the onset of CHK2 function in FOA at E18.5 coincided with the endpoint of the protective effect of AZT^18^, we tested if AZT treatment of *Chk2*^*−*/*−*^ mice prevented FOA completely. We validated that daily administration of AZT to pregnant females starting at E13.5 prevents FOA between E15.5 and E18.5, but fails to maintain this effect until P2 in control *Chk2*^*+/−*^ mice (Fig. 1d–g and Supplementary Data 1a, b)^18^. In contrast, AZT treatment of *Chk2*^*−/−*^ animals preserved more oocytes by P2 than either condition alone (Fig. 1f, g, i and Supplementary Fig. 3b and Supplementary Data 1a, b, e, f). In some cases, all fetal oocytes initially generated at E15.5 persist to P2 in *Chk2*^*−/−*^ + AZT conditions, resulting in a maximized oocyte supply (Fig. 1d–g). Notably, ending AZT treatment of *Chk2*^*−/−*^ mice at E18.5 did not maximize oocyte supply at P2 (Fig. 1f, h, i and Supplementary Data 1a, b).

### Mechanisms of AZT-sensitive FOA

Next, we wanted to identify the mechanism(s) of FOA that are independent of the CHK2-mediated DNA damage response and sensitive to AZT. We hypothesized that by inhibiting L1 reverse transcriptase activity with AZT, accumulation of intermediates of reverse transcription, such as RNA:DNA hybrids or single-stranded cDNA (ssDNA) during L1 retrotransposition attempts is diminished. Such nucleic acids are analogous to those produced by viruses, and when sensed by the host, can activate the innate immune system to eliminate infected cells. Indeed, L1 reverse transcription intermediates have been associated with activation of the host innate immune response, particularly the type I interferon pathway^45-47^.

To investigate whether the innate immune response was involved in AZT-sensitive FOA by targeting oocytes with high levels of L1 reverse transcription intermediates, we isolated and measured L1 ssDNA in untreated and AZT-treated oocytes and ovarian somatic cells (a negative control that does not express L1) (Supplementary Fig. 5a–c). To isolate ssDNA, we first treated total DNA with RNase A to remove residual mRNA and RNA within RNA:DNA hybrids. Subsequently, RNase A-treated DNA (input) was treated with double-stranded (ds) DNase to remove genomic DNA, leaving ssDNA (naked or once part of an RNA:DNA hybrid) (Supplementary Fig. 6a). To measure L1 ssDNA, quantitative PCR was performed to determine the abundance of L1 *ORF1* DNA in dsDNase-treated samples relative to input samples. The amount of L1 ssDNA is minimal relative to L1 input DNA considering that L1 is a repeat and comprises ~20% of the mouse genome. Indeed, we repeatedly observed a reduction of L1 *ORF1* ssDNA in AZT-treated oocytes compared to untreated oocytes (Supplementary Fig. 6b). Although this decrease was slight, the samples we compared are untreated oocytes containing sub-lethal levels of L1 activity and AZT-treated oocytes where L1 activity is suppressed, so extremely high levels of L1 ssDNA are not expected in either case. Importantly though, a large reduction in L1 *ORF1* ssDNA was observed in untreated ovarian somatic cells that do not express L1 compared to untreated oocytes that was not seen with corresponding AZT-treated samples (Supplementary Fig. 6b). From these data, we were encouraged to further explore a role for host immunity in FOA.

**Fig. 2:**
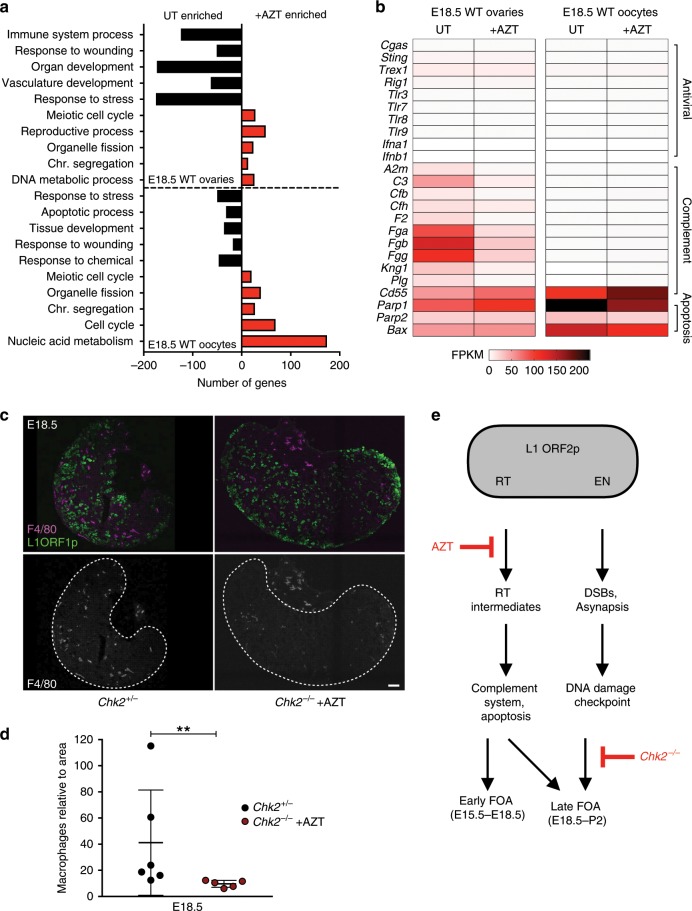
Mechanisms of AZT-sensitive FOA. **a** GO enrichment in untreated (UT) and AZT-treated E18.5 ovaries and oocytes. **b** mRNA abundance (FPKM) in UT and AZT-treated E18.5 ovaries and oocytes for immunity and apoptosis genes. **a, b** Analysis from mRNA-sequencing data containing *n* > 2 biological replicates sequenced per condition, each containing sorted oocytes or ovaries from > 6 WT CD1 embryos. **c** Immunofluorescence labeling of macrophage marker F4/80 and L1 ORF1p-labeling oocytes in E18.5 *Chk2*^+/−^ and *Chk2*^−/−^ + AZT ovaries. Ovaries are separated from surrounding tissue for quantification of macrophages in ovary with dotted line, boundary determined using L1 ORF1p as a marker of oocytes. Scale bar:50 μm. **d** Quantification of macrophage number per ovary section area in *Chk2*^+/−^ (*n* = 6) and *Chk2*^−/−^ + AZT (*n* = 5) conditions. Dots indicate independent ovary sections from three ovary samples per condition; data are mean + SD. Stats by Mann–Whitney test, **p < 0.01. **e** Model of L1 ORF2p activities and their relationship to molecular triggers and mechanisms of FOA. Reverse transcriptase (RT) activity of ORF2p is inhibited by AZT and associated with L1 reverse transcription intermediates, activation of the complement system and recruitment of immune cells. Endonuclease (EN) activity of ORF2p creates DNA breaks that in addition to other common meiotic defects, activates the DNA damage checkpoint through CHK2.

To determine whether the host innate immune system is involved in FOA, we analyzed oocyte autonomous (isolated oocytes) and non-autonomous (whole ovaries) gene expression profiles from wild-type untreated and AZT-treated mice at the onset and midpoint of FOA (E15.5 and E18.5, respectively) (Supplementary Fig. 7a, b). At E15.5, all oocytes should be present in untreated and AZT-treated conditions, but at E18.5, untreated samples contain surviving oocytes while AZT-treated samples contain rescued oocytes in addition to normally surviving oocytes. Pairwise comparison of untreated and AZT-treated E18.5 samples revealed the most significant differences related to FOA (Supplementary Fig. 7c–f and Supplementary Data 3a–d). When analyzing these differences by gene ontology enrichment, we found enrichment of immune pathways in untreated conditions compared to AZT-treated, thus linking immune pathways to FOA (Fig. 2a and Supplementary Data 6e–h). Interestingly, closer analysis of differential gene expression revealed antiviral innate immunity genes were not expressed in oocytes or ovaries of any condition, in contrast to studies that observe a relationship between such pathways and L1 expression in somatic cells (Fig. 2b and Supplementary Data 3i)^45-47^.

However, expression of genes involved in the complement system of innate immunity were differentially expressed between untreated and AZT-treated conditions. The complement system has been implicated in the adult ovary in response to follicular remodeling and results in recruitment of immune cells, such as macrophages during an inflammatory response^48^. Genes encoding molecules involved in complement system activation, such as *C3* are significantly increased in untreated compared to AZT-treated ovaries (Fig. 2b and Supplementary Data 3i). In contrast, expression of the *Cd55(Daf)* gene that protects cells from autologous complement attack was increased in E18.5 AZT-treated oocytes compared to untreated oocytes, suggesting that oocytes evading FOA are suppressing the complement system (Fig. 2b). We do not rule out that activation of the complement system may be either a direct response to L1 reverse transcription intermediates or an indirect response to the presence of dying oocytes as untreated oocytes also show increased expression of apoptotic genes such as *Parp1* and *Bax* (Fig. 2b). The relationship between complement system activation and FOA was further supported by observing a reduced number of macrophages in ovaries that have evaded FOA at E18.5 (Fig. 2c, d).

Cumulatively, our data suggests that FOA serves as quality control during the establishment of the ovarian reserve and identifies two major pathways driving FOA related to L1 activity, a prominent genotoxic threat during fetal oocyte development. First, L1 reverse transcriptase activity throughout meiotic prophase I results in accumulation of reverse transcription intermediates and ultimately activation of the complement system and recruitment of immune cells to eliminate fetal oocytes with excessive amounts of genotoxic stress (Fig. 2e). This mechanism of FOA is inhibited by AZT treatment. Second, the DNA damage checkpoint through CHK2 in the mid-pachytene stage of meiotic prophase I eliminates fetal oocytes as a result of excess DNA damage through L1 endonuclease activity and common meiotic defects (Fig. 2e). Importantly, we can now generate pups that have not experienced FOA using a combination of AZT treatment and *Chk2*^−*/−*^ and use this experimental system to study the biological relevance of FOA.

### Consequences of forgoing oocyte quality control

**Fig. 3:**
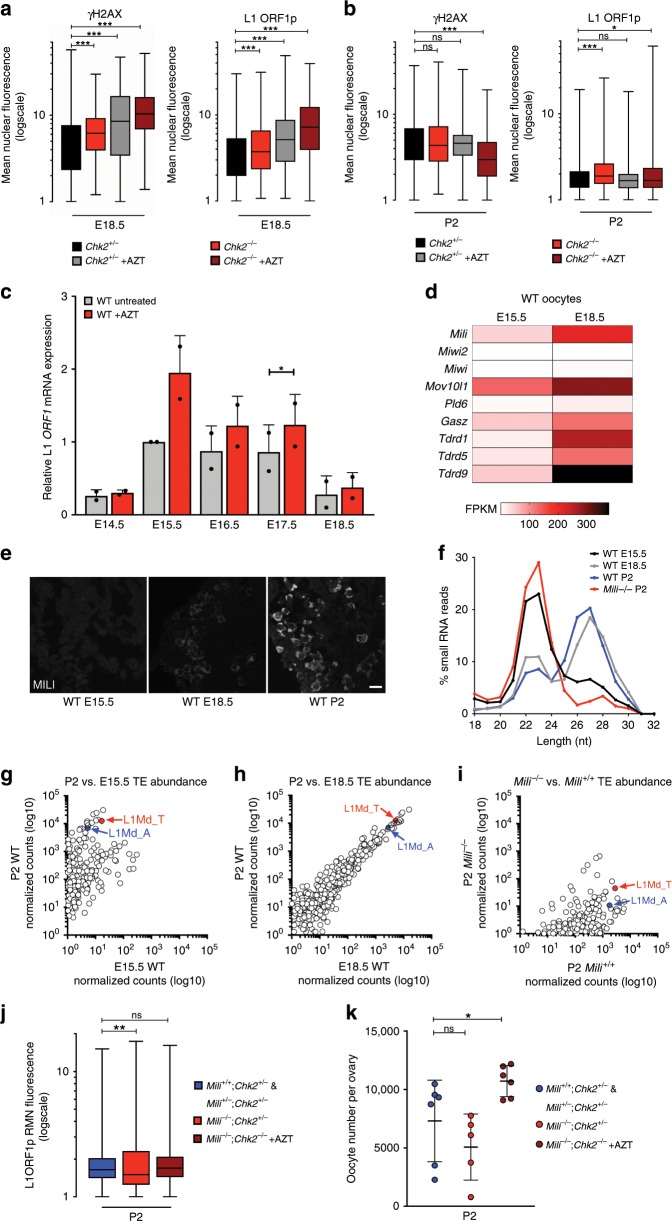
L1 genotoxicity is ultimately reduced in oocytes independent of piRNAs. **a**, **b** RMN fluorescence γH2AX and L1 ORF1p levels in *Chk2*^*+/−*^ and *Chk2*^*−*^^*/−*^ untreated and AZT-treated oocytes at **a** E18.5 and **b** P2. Box plots indicate center line at median value, box limits at upper and lower quartile values, and whiskers at max and min values; *n* > 118 oocytes per sample. Stats by Kolmogorov–Smirnov test, ns *p* > 0.05; *p < 0.05; ***p < 0.001. **c** L1 *ORF1* mRNA expression in UT and AZT-treated oocytes by qRT-PCR. Normalized to E15.5 UT with *Actb* internal control. *n* > 6 WT CD1 embryos from single litter per replicate, each bar represents two biological replicates. Dots indicate individual biological replicates; data are mean + SD. Stats by two-tailed paired Student’s *t*-test, *p < 0.05. Source data are provided as a Source Data file. **d** Expression of piRNA biogenesis genes in WT E15.5 and WT E18.5 sorted oocytes of CD1 genetic background. Two biological replicates per stage. **e** MILI expression in E15.5, E18.5, and P2 ovary sections of WT CD1 mice. Scale bars:15 μm. **f** Length distribution of 18–32nt small RNA reads from E15.5, E18.5, and P2 WT and P2 *Mili*^*−/−*^ ovaries. **g**–**i** Antisense transposon-targeting small RNAs in **g** E15.5 vs. P2 WT **h** E18.5 vs. P2 WT and **i** P2 *Mili*^*+/+*^ vs. P2 *Mili*^−*/−*^ ovaries. For small RNA-Seq, at least three pairs of ovaries from a single litter used per sample, data represents one biological replicate per sample. Experiment repeated for WT E18.5 and WT P2 conditions (Supplementary Data 4a). **j** RMN fluorescence L1ORF1p levels in *Mili*^*−/*^^*−*^*;Chk2*^*−/−*^ + AZT and control P2 ovaries. Box plots indicate center line at median value, box limits at upper and lower quartile values, and whiskers at max and min values; *n* > 127 oocytes per sample. Stats by Kolmogorov–Smirnov test, ns *p* > 0.05; **p < 0.01. **k** Oocyte number in *Mili*^*−/−*^*;Chk2*^*−*^^*/−*^ + AZT and control P2 ovaries. Dots indicate independent ovary samples; data are mean + SD; *n* > 3 ovaries per condition. Stats by two-tailed unpaired Student’s *t*-test, ns *p* > 0.05; *p < 0.05.

Our findings suggest that FOA serves as quality control for oocyte selection prior to birth. Therefore, we used our experimental system to inhibit FOA and ask if preserving oocytes with lethal amounts of genotoxic stress would be either detrimental for the ovarian reserve by reducing its overall quality, or potentially beneficial by increasing its size. To understand consequences to ovarian reserve quality by preserving oocytes with substantial genotoxic stress, we measured the nuclear abundance of γH2AX, which is a marker of common meiotic defects including unrepaired DSBs and chromosome asynapsis^49,50^, and L1 ORF1p in individual oocytes of *Chk2*^*+/−*^, *Chk2*^*−/−*^, and AZT-treated *Chk2*^*+/−*^ and *Chk2*^*−/*−^ ovaries. Consistent with previous findings^18^, a population of AZT-treated E18.5 *Chk2*^*+/−*^ or *Chk2*^*−/−*^ oocytes showed higher γH2AX and L1 ORF1p abundance never observed in untreated controls (Fig. 3a). Unexpectedly, P2 oocytes in all conditions showed significantly reduced γH2AX and L1 ORF1p levels compared to E18.5 (Fig. 3b and Supplementary Data 1g–j). Further, we find that reduction of L1 expression is initiated at the post-transcriptional level around E18.5 in both untreated and AZT-treated oocyte populations, despite higher levels of L1 RNA in AZT-treated oocytes at all stages between E14.5 and E18.5 (Fig. 3c). This FOA-independent reduction of L1 and γH2AX levels indicates that while FOA acts as quality control for genome integrity, given a chance, oocytes ultimately reduce genotoxicity.

### piRNA involvement during FOA and upon FOA evasion

Previous studies have shown significant DNA damage repair capacity of *Chk2*^*−/−*^ mutant postnatal oocytes^43^, but the unexpected reduction of L1, particularly at the post-transcriptional level, prompted us to examine the involvement of piRNAs in FOA. piRNAs are small PIWI-interacting RNAs that function to repress transposable elements by maintaining a methylated state or degrading transcripts. In males, piRNAs are essential for fertility; however, piRNA function in females is minimal and dispensable for fertility with the understanding behind this sexual dimorphism ambiguous. We wanted to understand a role for piRNAs in FOA and understand whether FOA is masking a potential relevance for piRNAs by eliminating oocytes with high levels of L1. Indeed, robust downregulation of L1 expression in E18.5-P2 oocytes coincided with increased expression of piRNA pathway genes including *Mili* that encodes the predominant Piwi family protein in oocytes (Fig. 3d, e)^51,52^. Consistent with piRNA pathway activation, RNAs of 26–27 nucleotides in length (characteristic of piRNA) became highly abundant in E18.5 and P2 wild-type ovaries and superseded RNAs of 22–23 nucleotides (characteristic of endo-siRNAs) dominating the E15.5 RNA profile (Fig. 3f and Supplementary Data 4a). Biogenesis of these piRNAs was Mili-dependent, as *Mili*^*−/−*^ P2 ovaries had negligible amounts of 26–27 nucleotides-long small RNAs compared to *Mili*^*+/+*^ (Fig. 3f and Supplementary Data 4a). By aligning small RNA reads to repetitive elements, a massive increase in antisense small RNAs targeting evolutionarily young L1MdA and L1MdT elements at E18.5 and P2 compared to E15.5 was observed, also in a *Mili*-dependent fashion (Fig. 3g–i and Supplementary Data 4b–d).

To test whether piRNAs produced in oocytes are involved in FOA, we generated *Mili*^*−/−*^*;Chk2*^*−/−*^ mice treated with AZT. These mice do not generate piRNAs nor undergo FOA. First, we quantified fluorescence intensity of L1 ORF1p at P2, when levels are normally reduced independently of FOA. We found that L1 ORF1p levels are also reduced by P2 in *Mili*^*−/*^^*−*^*;Chk2*^*−/−*^ + AZT oocytes and that piRNAs are not involved in FOA (Fig. 3j, k and Supplementary Data 5a–d). We now understand that the irrelevance of piRNAs in oocytes cannot be explained by FOA, and that other mechanisms to be discovered have evolved to reduce L1 in perinatal stages.

### Consequences of forgoing oocyte quality control

**Fig. 4:**
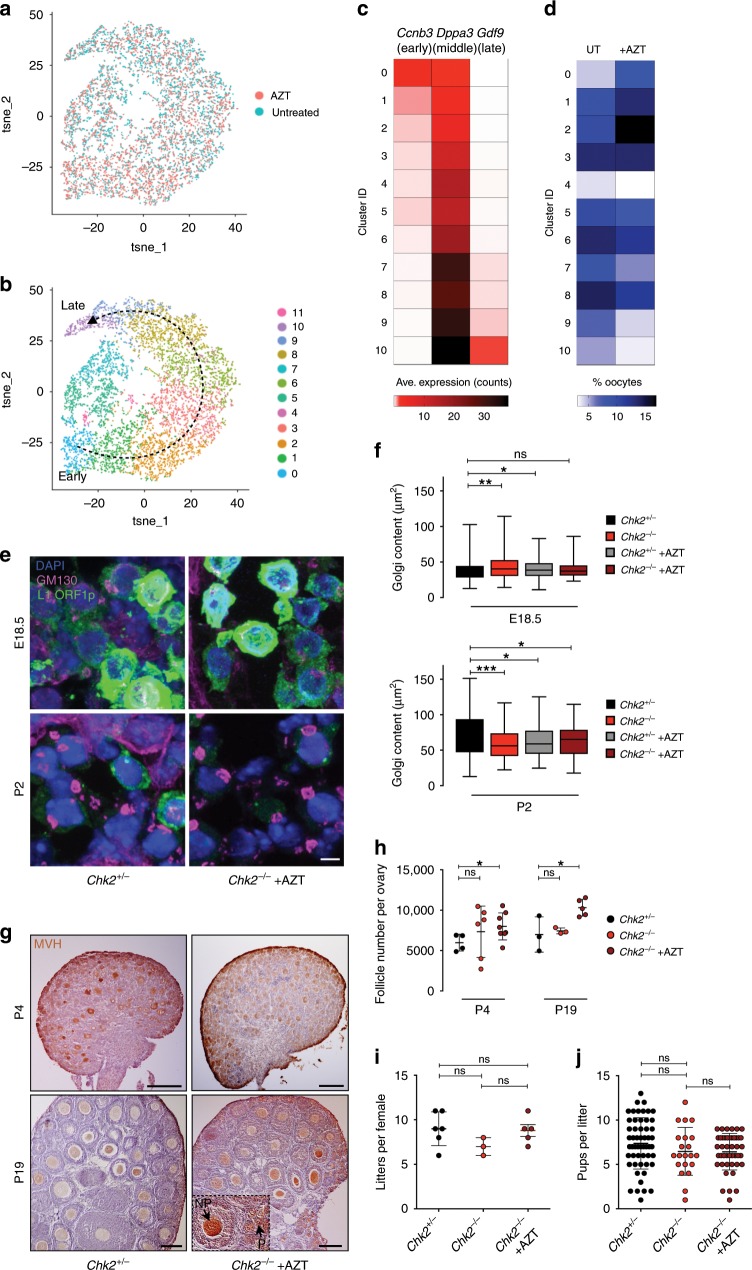
Oocyte differentiation and physiology are independent of FOA. **a** T-SNE plot displaying integrated E18.5 UT (blue) and AZT-treated (orange) oocytes colored by original identities. **b** T-SNE plot displaying cluster analysis of integrated E18.5 UT and AZT-treated oocytes. 11 clusters formed, cluster 11 removed from downstream analyses because top genes were mitochondrial, indicative of poor quality. Developmental trajectory based on known marker genes is indicated by arrows. **c** Heatmap of average expression of early (*Ccnb3*), middle (*Dppa3*), and late (*Gdf9*) marker genes within individual clusters. Clusters ordered from early to late developmental timepoints. **d** Percent of oocytes in untreated or AZT-treated samples belonging to each cluster, ordered from early to late developmental timepoints. Approximately 16,000 single-cells and 50,000 reads/cell collected from WT CD1 E18.5 untreated and AZT-treated ovaries from one litter of at least six pairs of ovaries each. Experiment repeated one time for both untreated and AZT-treated samples, but with ~5000 single-cells per sample and 7000–10,000 reads/cell (Supplementary Data 6c). **e** GM130 and L1 ORF1p expression in representative E18.5 and P2 *Chk2*^*+/−*^ and *Chk2*^*−/−*^ + AZT ovary sections. Scale bars: 5 μm. **f** Golgi area (μm^2^) in *Chk2*^*+/−*^ and *Chk2*^−*/*−^ untreated and AZT-treated E18.5 and P2 ovaries. Box plots indicate center line at median value, box limits at upper and lower quartile values, and whiskers at max and min values; *n* > 70 oocytes per sample. Stats by Kolmogorov–Smirnov test, ns *p* > 0.05; *p < 0.05; **p < 0.01; ***p < 0.001. **g** MVH and hematoxylin labeling of P4 and P19 *Chk2*^*+/−*^ and *Chk2*^*−/−*^ + AZT ovaries. Scale bars:100 μm. Inset exemplifies primordial (P) and non-primordial (NP) follicles. **h** Quantification of follicles in P4 and P19 ovaries. Dots indicate independent ovary samples; data are mean + s.e.m.; *n* > 3 ovaries per sample. **i** Number of litters from *Chk2*^*+/−*^, *Chk2*^*−*^^*/−*^, and *Chk2*^*−/−*^ + AZT females crossed to *Chk2*^*+/−*^ males. Dots indicate independent females; data are mean + SD; *n* > 3 females per condition. **j** Pups per litter from *Chk2*^*+/*−^, *Chk2*^*−*^^*/−*^, and *Chk2*^*−/−*^ + AZT females crossed to *Chk2*^*+/−*^ males. Dots indicate independent litters; data are mean + SD; *n* > 21 litters per condition. **h**–**j** Stats by two-tailed unpaired Student’s *t*-test, ns *p* > 0.05; *p < 0.01.

The striking reduction of L1 expression and repair of DNA breaks in oocytes evading FOA prompted us to test their differentiation and developmental capacity. We first used single-cell RNA sequencing to understand the composition and developmental progression of oocyte populations in untreated compared to AZT-treated conditions at E18.5 (Fig. 4a and Supplementary Fig. 8a, b). We subset oocytes from ovarian somatic cells based on expression of oocyte-specific genes *Ddx4, Dazl*, and *Mael*, in addition to the lack of *Xist* expression that was restricted to somatic cells. Then, we integrated the untreated and AZT-treated oocyte datasets and found that the two datasets overlay each other without any deviating populations specific to a particular sample. We performed cluster analysis on the integrated oocyte datasets and found that untreated and AZT-treated oocytes follow a similar developmental trajectory based on identified marker genes of early, middle, and late timepoints (Fig. 4a, b and Supplementary Fig. 8c and Supplementary Data 6a). However, upon comparing percent of untreated or AZT-treated oocytes belonging to each cluster, we find that early clusters are enriched for AZT-treated oocytes while late clusters are enriched for untreated oocytes (Fig. 4c, d and Supplementary Data 6b). We hypothesize that this shift is due to prevention of FOA in early stages, and sampling of oocytes for sequencing would enrich for this population.

To assess the differentiation potential of oocytes evading FOA, we next characterized the formation and growth of the Balbiani body, an organelle aggregate containing the Golgi complex, mitochondria, and RNAs that is a feature of oocyte differentiation^53–55^. Balbiani body sized based on Golgi content by immunofluorescence staining with the antibody GM130 increased from 40 μm^2^ at E18.5 to 70 μm^2^ at P2 in *Chk2*^*+/−*^-untreated oocytes, and similarly increased in *Chk2*^*−/−*^ untreated and *Chk2*^*+/−*^ and *Chk2*^*−/−*^ AZT-treated oocytes (Fig. 4e, f and Supplementary Data 1k, l). Therefore, oocytes allowed to reach P2 upon FOA evasion have the potential to differentiate.

Oocyte differentiation also requires surrounding individual oocytes with somatic granulosa and theca cells during follicle assembly. We wondered whether having an increased ovarian reserve impeded folliculogenesis or could not be sustained due to inadequate number of somatic cells and the demand for nutrients and growth signals derived from. We used immunohistochemistry to visualize follicle assembly from primordial to primary, secondary, to antral follicles, and quantify follicle number in normal conditions and upon FOA evasion. First, we observed P4 ovaries that are at the beginning stages of folliculogenesis, containing only primordial and primary follicles. We then compared these to P19 ovaries that have developed all follicles stages, but just prior to ovulation so no follicles have been lost. We observed all growing follicle types specific to P4 and P19 ovaries of *Chk2*^*+/−*^, *Chk2*^*−/−*^, and *Chk2*^*−/−*^ + AZT females (Fig. 4g). Importantly, quantification of primordial and non-primordial follicles revealed an increase in total follicles upon FOA evasion in P4 and P19 stages (Fig. 4h and Supplementary Data 7a, b). Therefore, increasing the oocyte supply by preventing FOA can increase the ovarian reserve into juvenile aged mice.

To test the effect of increased follicle number on fertility, we crossed females born to *Chk2*^*−/−*^ + AZT-treated females that have reached reproductive maturity to *Chk2*^*+/−*^ males and monitored the number of litters and litter size compared to untreated *Chk2*^*−/−*^ and *Chk2*^*+/−*^ females. Both litter number and litter size over 10 months was comparable between the three conditions, suggesting that FOA is not obligatory for fertility, nor is it detrimental to have an increased number of follicles in the ovarian reserve (Fig. 4i, j and Supplementary Data 7c–f).

## Discussion

In this work, we revisited a decades-old observation of FOA, a highly influential process of oocyte selection during the establishment of the mammalian ovarian reserve. We interrogated the striking correlation of FOA with a window of L1 retrotransposon expression and building upon previous studies, used L1 activities as a basis to understand the mechanisms and biological significance of FOA. A positive effect of the reverse transcriptase inhibitor AZT on fetal oocyte survival was previously identified, but was only effective until DNA damage checkpoint activation in mid-pachytene, E18.5 oocytes. We solved this limitation by perturbing the DNA damage checkpoint using *Chk2*^*−/*−^ in combination with AZT treatment, resulting in a maximized oocyte supply at birth. This elucidated two distinct mechanisms driving FOA, the canonical DNA damage checkpoint through CHK2 in late meiotic prophase I stages and a CHK2-independent mechanism that is sensitive to L1 reverse transcriptase activity in early meiotic prophase I stages. We used unbiased gene expression analyses to reveal mechanisms of FOA linked to L1 reverse transcriptase activity including the complement system and immune cell recruitment.

We continued to deepen our understanding of L1-driven FOA by analyzing the molecular triggers related to genotoxicity of L1 reverse transcriptase and endonuclease activities. By comparing untreated control oocytes to those we manipulate to evade FOA with AZT-treatment, we implicated L1 reverse transcription intermediates in oocyte death. Additionally, we showed by immunofluorescence that oocytes evading FOA also initially exceed the threshold of L1 expression and common meiotic defects including DNA damage allowed for survival. Cumulatively, our evidence supports the hypothesis that FOA acts as quality control for the genome to prevent transmission of damaged genetic material^56^. While proving a causal link between L1 and the DNA damage and other forms genotoxicity leading to FOA remains a challenge, L1 is a strong candidate given the evidence from this current study and prior studies that manipulate L1 using multiple different mouse models and observing a corresponding change in genotoxic stressors and FOA^18^.

Another important finding from our work is that fetal oocytes in the absence of repressive DNA methylation, and as we demonstrated, FOA and piRNAs, can still extinguish L1 expression. As cells go to great lengths to protect themselves from transposable elements, and have evolved diverse and numerous silencing mechanisms, which fetal oocytes are using is a fascinating open question. It has been described previously that piRNAs are non-essential for fertility in oocytes and FOA was used for an explanation, since oocytes with dangerous levels of L1 are already killed. We disproved this hypothesis, showing that L1 expression continues to decrease in *Mili*^*−/*^^*−*^*;Chk2*^*−/−*^ + AZT oocytes that do not generate piRNAs nor undergo FOA. Since piRNAs are involved in de novo methylation in male gonocytes, perhaps significant piRNA production in oocytes would lead to premature re-methylation and is therefore suppressed. We speculate that the RNAi pathway may be involved in downregulating L1 as endo-siRNA-length small RNAs were observed. However, the Dicer mutant phenotypes manifest at later stages of oogenesis^57^. Another attractive possibility is that some members of the extensive family of KRAB-ZFP proteins repress L1 in absence of other mechanisms^58,59^.

Finally, a major finding of this work was that folliculogenesis and fertility were not impacted by FOA prevention. Even up to 19 days old, the ovarian reserve remained significantly increased in AZT-treated *Chk2*^*−/−*^ females compared to controls. Therefore, our data support FOA as a non-obligatory program for oogenesis and that all oocytes generated have the potential to differentiate and are not fundamentally different in identity or developmental fate. This excludes hypotheses that require germ cell apoptosis for proper oogenesis, a developmental program exemplified in organisms such as *Drosophila* and *C. elegans*^60^. However, more precise methods allowing specific labeling of rescued vs. normally surviving oocytes and eventually offspring rather than enriching for them are necessary for this conclusion. Further support that FOA is non-essential comes from ex vivo studies of ESC or iPSC-derived germ cells combined with appropriate somatic cells that can form primordial follicle-like structures without any opportunity for FOA^61^.

Due to the conservation of FOA and L1 expression in human oocytes, whether our findings are of biomedical relevance for female fertility is an exciting future direction. Inhibiting L1 activity to prevent FOA is a potential avenue to improve premature ovarian failure by increasing the ovarian reserve. In light of evolution, however, fetal oogenesis is a critical window of opportunity for L1 to promote genetic diversity and adaptation to environmental stress, an influence that may outweigh the short-term consequences to the ovarian reserve size of an individual. Future studies analyzing retrotransposition frequency and mutations in a larger pedigree from *Chk2*^*−/−*^ + AZT females that did not experience FOA will answer these big questions of whether lack of quality control in fetal oogenesis manifests in the disease susceptibility or adaptive potential of future generations.

## Methods

### Animals

For this study, *Chk2*^*−/−*^ mice in a mixed C57Bl/6 and 129X1/Sv genetic background were used^44^. *Chk2*^*−/−*^ mice were backcrossed one time to C57Bl/6 to generate *Chk2*^*+/−*^ controls. We chose *Chk2*^*+/−*^ as a control to account for the mixed genetic background resulting in a significant increase in oocyte number compared to wild-type mice of pure C57Bl/6 background (Supplementary Fig. 1a–c). *Mili*^*+/+*^*, Mili*^*+/−*^, and *Mili*^*−/−*^ mice used were in C57Bl/6 genetic background. *Mili*^*−*^^*/−*^*;Chk2*^*−/−*^ mice and control littermates were generated by crossing *Chk2*^*−/−*^ and *Mili*^*+/−*^ animals. Wild-type mice of CD1 (Charles River Laboratories) genetic background were used for all quantitative PCR, mRNA (bulk ovary and oocyte and single-cell) and small RNA-sequencing experiments unless otherwise noted. All experimental procedures were performed in compliance with ethical regulations and approved by the Institutional Animal Care and Use Committee (IACUC) of the Carnegie Institution for Science under protocol number 157, “Evaluation of effects of transposon expression on the mouse germline”.

### AZT treatment

50 mg/kg/day AZT was administered daily by gavage to ~8-month-old pregnant female from E13.5 until experiment end point. AZT (Sigma Aldrich, Cat# A2169) was diluted to 5 mg/mL in nuclease-free water, aliquoted and stored at −20 °C. Short AZT treatment involved administration of 50 mg/kg/day AZT daily from E13.5 until E18.5, then no further treatment until P2 when ovaries were collected (Fig. 1f, h).

### Immunostaining

Whole-mount immunofluorescence was performed on ovaries that had been fixed for 2 h in 2% paraformaldehyde followed by three washes in 1X PBS, 30 min each, a wash in 10% and 20% sucrose each for one hour at 4 °C and 30% sucrose overnight at 4 °C. Ovaries were then permeabilized in 0.5% Triton X-100–1X PBS for 4 h at 4 °C followed by incubation in blocking solution containing 10% normal serum and 3% BSA overnight at 4 °C. Primary antibody was diluted in blocking solution and ovaries incubated for 2 days at 4 °C. Ovaries were washed three times, 30 min with 0.1% Triton X-100 in 1X PBS at room temperature. Secondary antibody was diluted in blocking solution and ovaries were incubated overnight at 4 °C. Ovaries were washed three times, 30 min with 0.1% Triton X-100 in 1X PBS at room temperature, then replaced solution with ScaleA2 clearing reagent. ScaleA2 was replaced daily for 7 days, then ovaries were imaged by confocal microscopy. For immunofluorescence on ovary sections, ovaries were fixed for 2 h in 2% paraformaldehyde followed by three washes in 1X PBS, 30 min each, a wash in 10% and 20% sucrose each for one hour at 4 °C and 30% sucrose overnight at 4 °C. Ovaries were sectioned into 8 μm slices on poly-lysine-coated slides. Slides were washed for 5 min with 1X PBS and permeabilized in 0.1% Triton X-100 for 10 min. Slides were then incubated in blocking solution containing 10% normal serum for 30 min at room temperature. Primary antibody was diluted in blocking solution with 3% normal serum and slides incubated overnight at 4 °C. Slides were washed for 10 min with 0.1% Triton X-100 in 1X PBS followed by two washes for 10 min with 0.05% Triton X-100 in 1X PBS. Secondary antibody was diluted in blocking solution with 3% normal serum and slides incubated for 2 h at room temperature. Slides were washed for 10 min with 0.1% Triton X-100 in 1X PBS followed by two washes for 10 min with 0.05% Triton X-100 in 1X PBS. DAPI was added and slides washed in 1X PBS and mounted using Prolong Diamond antifade mountant. For immunohistochemistry with DAB staining, ovaries were fixed in Bouin’s overnight at 4 °C, transferred to 70% ethanol overnight, and embedded in paraffin. Ovaries were sectioned into 10 μm slices. Sections were deparaffinized by washing slides in Citrisolv (Decon Labs) 3× 15 min, re-hydrated through graded ethanol washes, blocked with hydrogen peroxide for 10 min, avidin block for 15 min (Vector Laboratories), biotin block (Vector Laboratories), and goat serum block (Vector Labs cat #PK-4001). Samples were incubated overnight at 4 °C with anti-DDX4/MVH and for 30 min at room temperature with biotinylated goat anti-rabbit IgG secondary antibody (Vector Labs cat #PK-4001). Samples were incubated 30 min at room temperature with Vectastain ABC reagent (Vector Labs cat #PK-4001) followed by DAB detection. Slides were dipped in hematoxylin, rehydrated in ethanol, dipped in Citrisolv and mounted.

### Antibodies

The following primary antibodies and dilutions were used: anti-Germ cell-specific antigen–antibody [TRA98], rat monoclonal, abcam cat. #ab82527, diluted to 1:500 for immunofluorescence; anti-L1 ORF1p (full length protein), rabbit polyclonal, diluted to 1:500 for immunofluorescence ^18^; anti-GM130, mouse monoclonal, BD Biosciences cat. #610822, diluted to 1:200 for immunofluorescence; anti-phospho-Histone H2A.X (Ser139) clone JBW301, mouse monoclonal, Millipore Sigma cat. #05-636, diluted to 1:1000 for immunofluorescence; anti-SYCP3, rabbit polyclonal, abcam cat. #ab15093, diluted to 1:500 for immunofluorescence. Anti-PIWIL2, rabbit polyclonal, abcam cat. #ab181340, diluted to 1:50 for immunofluorescence; anti-F4/80 antibody [CI:A3-1], rat monoclonal, abcam cat. #ab6640, diluted to 1:100 for immunofluorescence; anti-DDX4/MVH, rabbit polyclonal, abcam cat. #ab13840, diluted to 1:200 for immunostaining on paraffin sections and 1:1000 for western blot; anti-p63(4A4), mouse monoclonal, Santa Cruz Biotechnology cat. #sc-8431, diluted to 1:500 for western blot. The following secondary antibodies and dilutions were used: Alexa donkey ant-rabbit 488 (Invitrogen, cat #A-21206) diluted 1:1000 for immunofluorescence; Alexa donkey anti-rabbit 568 (Invitrogen, cat #A10042) diluted 1:1000 for immunofluorescence; Alexa donkey anti-mouse 488 (Invitrogen, cat #A-21202) diluted 1:1000 for immunofluorescence; Alexa donkey anti-mouse 594 (Invitrogen, cat #A-21203) diluted 1:1000 for immunofluorescence; Alexa donkey anti-rat 647 (Invitrogen, cat #150155) diluted 1:1000 for immunofluorescence; Goat anti-mouse IgG (H + L)-HRP Conjugate (BioRad cat #1721011) diluted 1:2000 for Western blot; Goat anti-rabbit IgG (H + L)-HRP conjugate (BioRad cat #1721019) diluted 1:2000 for Western blot.

### Microscopy

Imaging of whole-mount ovaries and ovary sections was performed using TCS-SP5 laser-scanning confocal microscope (Leica, Buffalo Grove, IL), histological sections using Nikon Eclipse E800 microscope equipped with a Diagnostic Instruments model 2.3.1 digital camera, and meiotic spreads using Olympus BX61 microscope equipped with a Hamamatsu C4742-95 digital Camera. Image analysis was completed using Imaris (Bitplane) and ImageJ.

### Oocyte and follicle quantification

We quantified oocytes per ovary at E15.5, E18.5, and P2 using a whole-mount immunofluorescence and tissue clearing method explained in detail in the “Immunostaining” section of “Methods”^62^. Ovaries were dissected and labeled with the germ cell-specific antibody TRA98^63^. After immunofluorescence, samples were treated with ScaleA2 clearing reagent for 7 days, changing solution each day. Confocal imaging through the entire tissue using SP5 confocal microscope (Leica) followed by 3D reconstruction of Z-stacked images and spot and surface analysis using Imaris software (Bitplane) were performed. For statistical analysis, average number of oocytes per ovary was counted in ovaries from at least three different embryos from 1 to 4 litters (Supplementary Data 1a, c). Variability between numbers due to timing of plug during the day as well as natural variation between embryos that is more apparent at earlier stages between litters and embryos. Statistical significance was determined using two-tailed unpaired Student’s *t*-test (Supplementary Data 1b, d). We also quantified oocytes using sectioning-based methods and immunofluorescence. Ovary sections of 8 μm thickness were labeled with germ cell-specific antibody TRA98 and nuclei counterstained with DAPI. Oocytes were counted in every 5th section through the entire ovary. Using these numbers, we estimated the total oocyte number per ovary. Statistical significance was determined using two-tailed unpaired Student’s *t*-test (Supplementary Data 1e, f and 5a, b). We quantified primordial and non-primordial follicles at P4 and P19 by performing immunohistochemistry and DAB staining on paraffin-embedded ovarian sections. Ovary sections of 10 μm thickness were labeled with the cytoplasmic germ cell marker MVH and nuclei counterstained with hematoxylin. MVH-positive follicles were quantified in every 5th section through the entire ovary and categorized as primordial or non-primordial based on the number of somatic cell layers surrounding the oocyte. Total follicle number per ovary was estimated based on these counts. At least three ovaries from three different females were quantified for each experimental group and two-tailed unpaired Student’s *t*-test was used to determine statistical significance (Supplementary Data 7a, b).

### Analysis of L1ORF1p and γH2AX nuclear fluorescence

Ovary cryosections of 8 μm thickness were stained with DAPI, germ cell marker TRA98, and L1 ORF1p. Confocal stacks were taken through the section. Imaris bitplane was used to generate a surface around each DAPI-positive nucleus in TRA98-positive germ cells. Then, relative mean nuclear (RMN) fluorescence was calculated for the channel containing L1 ORF1p signal within the surface. This procedure was used in the same manner to calculate γH2AX RMN fluorescence. Each germ cell RMN value was then divided by the average of three RMN values from TRA98-negative somatic cell nuclei that should not contain L1 ORF1p nor γH2AX to normalize for background fluorescence. On average, about 200 oocytes were quantified per experimental group. Oocytes come from at least three different ovaries and two different litters unless noted otherwise (Supplementary Data 1g, i, and Supplementary Data 5c). Statistical significance was determined using two-tailed Kolmogorov–Smirnov test (Supplementary Data 1h, j and Supplementary Data 5d).

### Analysis of Golgi element area

Ovary cryosections of 8 μm thickness were stained with DAPI, germ cell markers TRA98 and GM130^24^. Confocal stacks were taken through the section. Imaris bitplane was used to generate a surface around each GM130-positive Balbiani body region in a TRA98-positive cell. Area of surface generated for GM130 channel was calculated. Each bar represents 70–200 individual oocytes measured. Oocytes come from at least three different ovaries and two different litters unless noted otherwise (Supplementary Data 1k). Statistical significance was determined using two-tailed Kolmogorov–Smirnov test (Supplementary Data 1l).

### Meiotic chromatin spread preparation

Ovaries were dissociated into a single-cell suspension using dissociation buffer containing 0.025% trypsin, 2.5 mg/mL collagenase, and 0.1 mg/mL DNase I. One volume of hypotonic buffer (30 mM Tris–HCl, pH 8.2, 50 mM sucrose, 17 mM sodium citrate) was added to the cell suspension and set on nutator for 30 min. Cells were pelleted and supernatant replaced with 100 μM sucrose, pH 8.2 solution. Approximately 600 μL sucrose solution per ovary pair. Slides were dipped in fixative (1% PFA, 0.15% Triton X-100, pH 9.2) and 20 μL resuspended cells pipetted along bottom edge. Cells were slowly spread around slide by tilting the slide gently. Slides were dried in humid chamber for 2 h, then treated with 0.08% Photo-Flo (Kodak). Slides used immediately for immunostaining or stored at −80 °C.

### Western blot

6–12 WT whole ovaries were lysed in RIPA buffer containing 50 mM Tris–HCl pH = 8, 150 mM NaCl, 1% NP40, 1% SDS, 1 mM EDTA, and 10% glycerol. 1 mM PMSF and 1 mM Halt protease and phosphatase inhibitor cocktail were added to buffer just before lysis. Ovaries were homogenized using RNase-free pestle and protein quantified using BCA. Lysates were run on 12% polyacrylamide running, 4% polyacrylamide stacking gel. Proteins transferred overnight at 4 °C to PVDF membrane that had been activated for 15 s in 100% methanol followed by 2 min water and 15 min in transfer buffer. Membrane rinsed with PBS + 0.05% Tween-20 and blocked with 5% nonfat milk in PBS + 0.05% Tween-20 for 1 h at room temperature. Primary antibodies were incubated overnight at 4 °C in blocking buffer. Secondary antibodies used at 1:2000 and incubated for 1 h at room temperature. Detection by ECL was performed.

### FACS sorting

To isolate oocytes for sequencing and q-PCR experiments, ovaries were dissociated into a single-cell suspension using dissociation buffer containing 0.025% trypsin, 2.5 mg/mL collagenase, and 0.1 mg/mL DNase I. Cell suspensions were filtered using 40 μm filter. Oocytes were FACS-sorted from remaining ovarian somatic cells based on size and complexity using forward and side scatter parameters (Supplementary Fig. 5a–c)^64^. Propidium iodide treatment for 10 min and gating for negative red fluorescence was used to eliminate dead cells from the sort. BD FACS Aria III sorter was used.

### L1 reverse transcription intermediates

Sorted oocytes or somatic cells from >6 pairs of E16.5–E17.5 WT CD1 untreated or AZT-treated ovaries from a single litter per sample were collected and treated with lysis buffer (100 mM Tris pH 8.5, 50 mM NaCl, 5 mM EDTA, 0.2% SDS) containing Proteinase K for 2 h at 55 °C. Lysate was treated with RNase A for 30 min at 37 °C to remove mRNA and RNA from RNA:DNA hybrids, and followed by DNA extraction using phenol:chloroform pH = 8 and salt/isopropanol precipitation. DNA abundance was measured using qBIT hsDNA system. Approximately 25 ng used as input for downstream reactions. RNase A-treated DNA (input) was exposed to either dsDNase to isolate ssDNA, ssDNase (P1) + dsDNase to eliminate all DNA, or P1 alone to isolate dsDNA. P1 nuclease (NEB, cat #M0660S) reactions were performed in 10 µL volume, incubated at 37 °C for 30 min followed by inactivation at 75 °C for 10 min. dsDNase (Thermofisher, cat #EN0771) reactions were performed in 20 µL volume, incubated at 37 °C for 10 min followed by inactivation at 55 °C for 5 min with addition of 10 mM DTT. Resulting samples were diluted equally and used for q-PCR detection of L1 *ORF1* and single-copy gene *Ifnb1*. Relative quantities from q-PCR are normalized to *Ifnb1* input relative quantity to account for total DNA concentration across samples. Then, resulting relative quantities are normalized to L1 *ORF1* input for respective sample. Five biological replicates used for untreated and AZT-treated, dsDNase-treated oocyte DNA, three biological replicates for untreated and AZT-treated, dsDNase-treated somatic cells, two biological replicates for untreated and AZT-treated, P1-treated oocyte DNA, and P1 + dsDNase-treated oocyte DNA. Statistical significance determined using two-tailed paired Student’s *t*-test for comparison of untreated to AZT-treated samples and Mann–Whitney test for comparison of WT untreated and AZT treated, dsDNase-treated oocytes to WT untreated and AZT-treated negative controls.

### Quantitative RT-PCR

Sorted oocytes or somatic cells from >6 pairs of WT CD1 untreated or AZT-treated ovaries from a single litter per sample were isolated at all stages between E14.5 and E18.5. RNA was extracted from cells using TRIZOL reagent (Invitrogen). RNA was DNase treated using TURBO DNA-free kit (Ambion). cDNA synthesis reactions were performed using oligo dT and Superscript III First-Strand Synthesis System for RT-PCR (Invitrogen). No reverse transcriptase controls were performed side-by-side. cDNA was diluted equally and added to the qRT-PCR reactions containing SsoAdvanced Universal SYBR Green Supermix (Bio-Rad). qRT-PCR was performed on CFX96 Touch Real-Time PCR Detection System to detect SYBR Green. Relative quantities were analyzed using ΔΔCt methods with *Actb* as the housekeeping control gene. Statistical significance was determined using two-tailed paired Student’s *t*-test.

### Primer sequences

Primer sequences used for qPCR and qRT-PCR experiments include: F-L1ORF1: ATG GCG AAA GGT AAA CGG AG; R-L1ORF1: AGT CCT TCT TGA TGT CCT CT; F-ifnb1: CTG CGT TCC TGC TGT GCT TCT CCA; R-ifnb1: TTC TCC GTC ATC TCC ATA GGG ATC; F-actb: CGG TTC CGA TGC CCT GAG GCT CTT; R-actb: CGT CAC ACT TCA TGA TGG AAT TGA; F-mvh: TGG CAG AGC GAT TTC TTT TT; R-mvh: CGC TGT ATT CAA CGT GTG CT.

### mRNA sequencing

WT CD1 whole ovaries or sorted oocytes were obtained. At least three pairs of ovaries from a single litter or oocytes sorted from at least six ovary pairs from a single litter were used per biological replicate. Two biological replicates were used for each whole ovary sample (E15.5 and E18.5 untreated and AZT-treated samples), two biological replicates were used for each sorted oocyte-untreated sample (E15.5 and E18.5), and three biological replicates used for each sorted oocyte AZT-treated sample (E15.5 + AZT and E18.5 + AZT). RNA was extracted from samples using Trizol reagent (Invitrogen), DNaseI-treated using TURBO DNA-free kit (Ambion), and libraries generated using ribo-zero kit. 75 bp unpaired, single-end reads were sequenced on Illumina Next-seq 500 system. Any remaining rRNA sequences were removed computationally using Bowtie by aligning reads to mm10 rRNA genome. Non-rRNA reads were subsequently mapped to mm10 genome using Tophat splice aligner^65^. To determine differential gene expression, cuffdiff was used followed by cummeRbund in R to obtain FPKM values and generate plots (Figs. 2b, 3d, Supplementary Fig. 7a–f and Supplementary Data 3a–d, i). GO pathway-enrichment analyses was performed using DAVID Bioinformatics Resources 6.8 (Fig. 2a, Supplementary Data 3e–h) ^66,67^.

### Small RNA sequencing

Total RNA was extracted from whole ovaries using mirVana miRNA isolation kit. RNA was run on 15% urea gel and 18–35 nucleotide region excised. Small RNAs were eluded from gel slice with 0.3 M NaCl overnight at room temperature. 3′ and 5′ adapters were added and reverse transcription reaction performed to generate cDNA. Libraries were PCR amplified and 75 or 150 bp reads sequenced on Illumina Next-Seq 500 system. At least three pairs of ovaries from a single litter were used per biological replicate. Data shown represents one biological replicate per condition. Experiment repeated for WT E18.5 and WT P2 conditions (Supplementary Data 4a). piPipes small RNA analysis pipeline was used to determine small RNA length distribution and to align reads to repeats (Supplementary Data 4a–d)^68^. First, we computationally removed adapter sequences and piPipes removes reads mapping to rRNA. Following this, piPipes aligns remaining reads to miRNA hairpins described in miRbase. These reads were removed, and used for normalization after aligning remaining non-rRNA, non-miRNA reads to the provided genome (mm10) using Bowtie. For all small RNA-seq experiments, at least three pairs of ovaries were used per sample. WT samples were of CD1 genetic background. *Mili*^*+/+*^ and *Mili*^*−/−*^ were of B6 genetic background. Shown are results from single replicates.

### Single-cell RNA sequencing

At least six whole ovaries from a single litter were dissociated into a single-cell suspension using dissociation buffer containing 0.025% trypsin, 2.5 mg/mL collagenase, and 0.1 mg/mL DNase I. Cells were pelleted and washed with PBS three times. Viability and cell count were determined using trypan blue staining and Countess II automated cell counter. Samples with >90% viability and 1 million cells per milliliter were used for sequencing. GEM generation, barcoding, and library construction were performed with 10x genomics Chromium Genome Reagent Kit (v2 Chemistry). Libraries were sequenced on Illumina Next-Seq 500 system. For the untreated sample, 16,448 cells with 48,924 mean reads per cell were sequenced. For the AZT-treated sample, 15,551 cells with 48,450 mean reads per cell were sequenced. Experiment replicated for both UT and AZT-treated ovaries using fewer cells (~5000 cells per sample) and fewer reads (7000–10,000 reads per cell) (Supplementary Data 6c). Differential gene expression and clustering analysis was performed using Cell Ranger v3.0 and Seurat v3.0 packages (Supplementary Data 6a, b)^69,70^. Untreated and AZT-treated oocyte are subset from total ovarian cells based on expression of *Ddx4, Dazl*, and *Maelstrom* and lack of expression of *Xist*. Oocyte datasets are integrated and cluster analysis performed. Oocyte clusters are ordered from those containing early, middle, and late stage oocytes based on marker gene expression (*Ccnb3* = early, *Dppa3* = middle, and *Gdf9* = late, expression values represented as Ave log FC). Percent of oocytes belonging to untreated and AZT-treated samples were calculated for each cluster.

### Fertility assay

*Chk2*^*−/−*^ females that were treated with AZT during their fetal development and raised to adults were crossed to *Chk2*^*+/−*^ males. *Chk2*^*−/−*^ females that were untreated as well as *Chk2*^*+/−*^ females that were untreated were crossed to *Chk2*^*+/−*^ males for controls. The number of litters produced over 10 months per female were reported for females that survived the duration of the assay (Supplementary Data 7c, d). The number of live pups per litter at the day of birth from 6 *Chk2*^*−/−*^ + AZT females, 3 *Chk2*^*−*^^*/−*^ females, and 6 *Chk2*^*+/−*^ females were monitored for at least 10 months (Supplementary Data 7e, f). Statistical significance was determined using unpaired Student’s *t*-test (Supplementary Data 7d, f).

### Statistical information

Two-tailed unpaired Student’s *t*-test used for oocyte quantification and fertility assays. Two-tailed paired Student’s *t*-test used for quantitative PCR comparing L1 *ORF1* DNA between WT untreated and WT + AZT samples (Supplementary Fig. 6b) and quantitative RT-PCR (Fig. 3c). Mann–Whitney test used for macrophage analysis (Fig. 2d) and for quantitative PCR comparing L1 *ORF1* DNA between WT-untreated dsDNase (oocyte) and WT-untreated negative controls (Supplementary Fig. 6b). Two-tailed Kolmogorov–Smirnov test was used for all RMN fluorescence experiments. Chi-square test was used for meiotic progression analysis. Statistics was calculated using GraphPad Prism 7 Software.

### Reporting summary

Further information on research design is available in the Nature Research Reporting Summary linked to this article.

## Supplementary information

Supplementary Information

Description of Additional Supplementary Files

Supplementary Data 1

Supplementary Data 2

Supplementary Data 3

Supplementary Data 4

Supplementary Data 5

Supplementary Data 6

Supplementary Data 7

Reporting Summary

## Data Availability

RNA sequencing data generated in this study have been submitted to the NCBI Sequence Read Archive project number PRJNA543598. The source data underlying Fig. 3c, Supplementary Figs. 4a and 6b are provided as a Source Data file. Additional relevant data are available from the authors upon request.

## Source Data

Source Data

## Code availability

Code used to obtain data throughout the manuscript is available from the authors upon request.
