# Supplementary material for: Maximizing the ovarian reserve in mice by evading LINE-1 genotoxicity
Source: Nat Commun. 2020 Jan 16;11:330. doi: 10.1038/s41467-019-14055-8 (PMC6965193; doi:10.1038/s41467-019-14055-8)
Supplement: Supplementary file 1 — Supplementary Information [file 41467_2019_14055_MOESM1_ESM.pdf]

Supplementary Information

Maximizing the ovarian reserve by evading LINE-1 genotoxicity

Tharp et al.

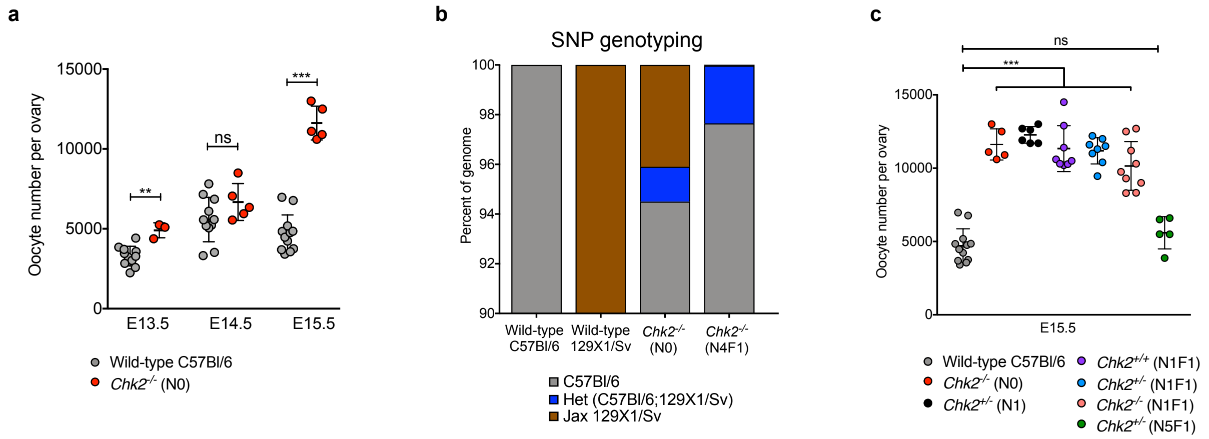

Supplementary Figure 1: Genetic background influences maximum oocyte number. (a) We observed a significant difference in maximum oocyte number per ovary at E15.5 and at earlier stages in *Chk2*$^{-/-}$ (N0) mice used for all other experiments and wild-type C57Bl/6 mice. Although appearing C57Bl/6, *Chk2*$^{-/-}$ (N0) mice were of mixed C57Bl/6 and 129X1/Sv genetic backgrounds. **(b)** We performed SNP genotyping (DartMouse, Dartmouth School of Medicine) to assess the amount of 129X1/Sv specific polymorphisms remaining in the *Chk2*$^{-/-}$ (N0) genome to determine whether genetic background contributed to the difference in maximum oocyte number observed, or whether this difference was related to CHK2 function[25]. Shown is percent genome of *Chk2*$^{-/-}$ (N0) mice belonging to C57Bl/6 or Jax 129X1/Sv origin, alongside wild-type C57Bl/6 and wild-type 129X1/Sv genomes that contain 100% of SNPs from respective origins. Y-axis is from 90 to 100%. Approximately 4% of the *Chk2*$^{-/-}$ (N0) genome was homozygous 129X1/Sv. We repeated SNP genotyping after backcrossing *Chk2*$^{-/-}$ (N0) to wild-type C57Bl/6 four times (N4F1), finding that homozygous 129X1/Sv SNPs are eliminated. (**c**) Maximum oocyte number per E15.5 ovary from respective genotypes and genetic backgrounds: wild-type C57Bl/6, *Chk2*$^{-/-}$ (N0), *Chk2*$^{+/-}$ (N1), *Chk2*$^{+/+}$ (N1F1), *Chk2*$^{+/-}$ (N1F1), and *Chk2*$^{-/-}$ (N1F1), and *Chk2*$^{+/-}$ (N5F1). *Chk2*$^{-/-}$ (N0) and *Chk2*$^{+/-}$ (N1) data are repeated from Fig. 1d and Supplementary Table 1a, b. Dots indicate independent ovary samples; data are mean $\pm$SD; n$\geq$3 ovaries. Stats by two-tailed unpaired Student's t-test, ns p>0.05; ***p<0.001. Only in *Chk2*$^{+/-}$ (N5F1), after five backcrosses to C57Bl/6, is the maximum oocyte number comparable to that of wild-type C57Bl/6, indicating that maximum oocyte number is dependent on genetic background (Supplementary Table 1c, d). Similar oocyte numbers observed between *Chk2*$^{+/+}$ (N1F1), *Chk2*$^{+/-}$ (N1F1), and *Chk2*$^{-/-}$ (N1F1), indicating that the difference in maximum oocyte number is not due to CHK2 function. For all other experiments, we compared *Chk2*$^{-/-}$ (N0) to *Chk2*$^{+/-}$ (N1) as a control rather than wild-type C57Bl/6 to avoid differences in oocyte number related to genetic background while still being able to obtain 100% progeny of desired genotype using the following crosses: WT B6 x *Chk2*$^{-/-}$ (N0) for the *Chk2*$^{+/-}$ control group and *Chk2*$^{-/-}$ (N0) x *Chk2*$^{-/-}$ (N0) for the *Chk2*$^{-/-}$ experimental group.

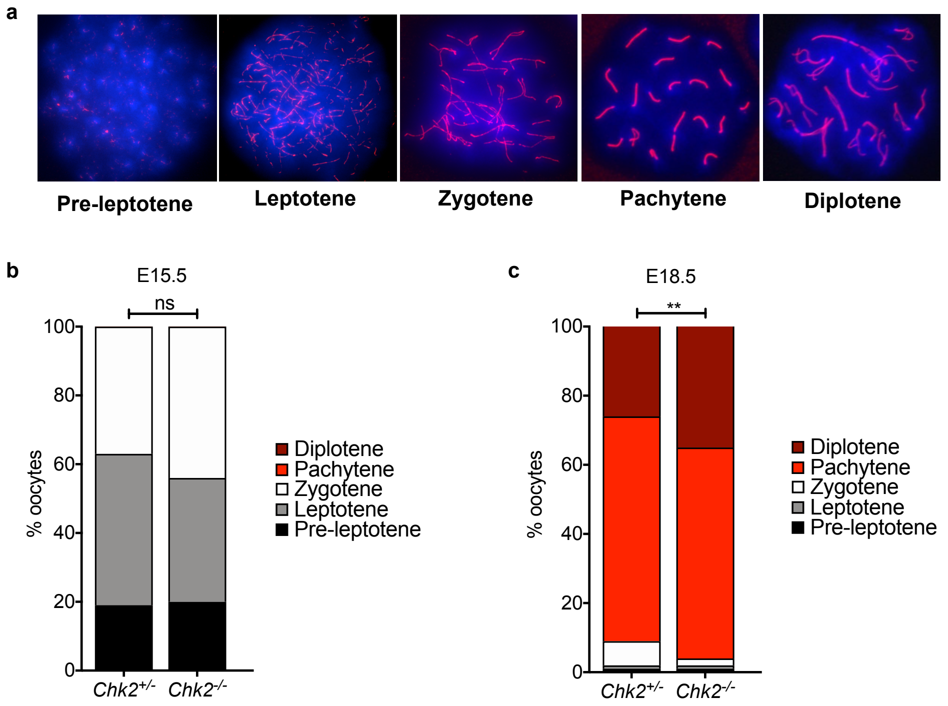

Supplementary Figure 2: Meiotic progression in the absence of *Chk2*. (a) Representative images of pre-leptotene, leptotene, zygotene, pachytene, and diplotene stages of meiotic prophase I. Synaptonemal complex labeled in red (SYCP3) and nucleus counterstained with DAPI. **(b, c)** Comparison of meiotic progression between *Chk2*$^{+/-}$ and *Chk2*$^{-/-}$ ovaries at (**b**) E15.5 (**c**) E18.5. Stats by Chi-square test, ns $p>0.05$, $^{**}p<0.01$ (Supplementary Table 2a, b).

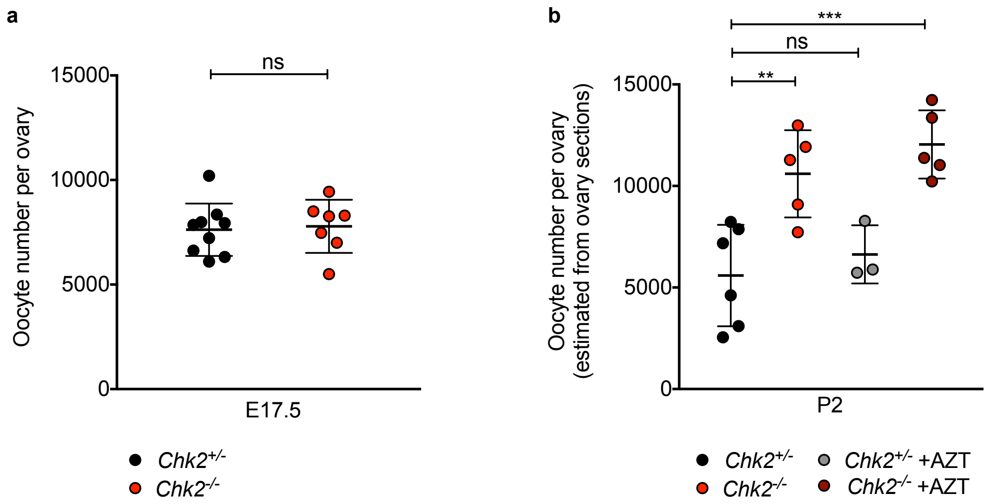

Supplementary Figure 3: Additional stages and replicates to understand FOA dynamics in the absence of *Chk2*. (a) Oocyte number in *Chk2*$^{-/-}$ and *Chk2*$^{+/-}$ ovaries at E17.5, an additional stage to E18.5 that is prior to DNA damage checkpoint activation. **(b)** Oocyte number at P2 in untreated and AZT-treated *Chk2*$^{+/-}$ and *Chk2*$^{-/-}$ ovaries using alternative oocyte quantification method. Oocyte number per ovary was estimated by counting oocytes in every 5th section throughout an entire ovary. The trend observed in FOA dynamics replicates that observed using whole-mount oocyte quantification method in Fig. 1d-g. Raw oocyte numbers slightly differ due to the estimation method in sections vs. counting each oocyte in the whole-mount method. Stats by two-tailed unpaired Student's t-test, ns $p>0.05$; **$p<0.01$; ***$p<0.001$. Dots indicate independent ovary samples; data are mean $\pm$ SD; $n\geq3$ ovaries per sample from at least 3 different embryos of two litters (a) or a single litter (b).

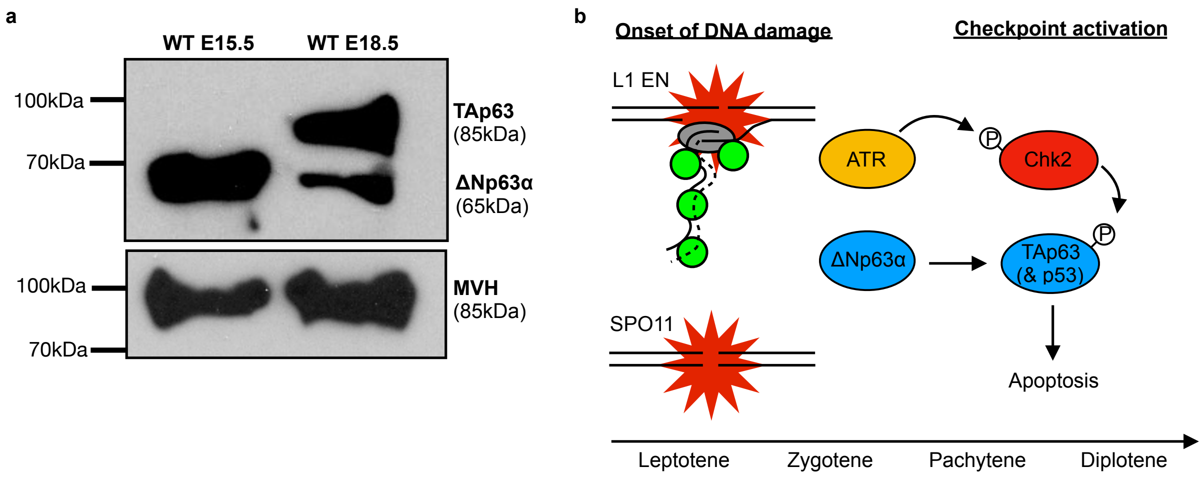

Supplementary Figure 4: Differential expression of p63 isoforms upon activation of the DNA damage checkpoint. (a) Western blot detection of p63 dominant negative (ΔNp63) and transactivating (TAp63) isoforms in E15.5 and E18.5 ovary lysates of wild-type C57Bl/6 mice. Western blot detection of mouse vasa homolog (MVH) in same lysates as a loading control. (**b)** Model of the DNA damage response mediated by checkpoint kinase 2 (CHK2). The presence of unrepaired DNA breaks generated during leptotene, zygotene, and early pachytene stages by L1 endonuclease (EN) activity or for meiotic recombination by Spo11 is relayed to CHK2 by ataxia telangiectasia and Rad3-related protein (ATR) through phosphorylation (P). CHK2 subsequently phosphorylates the active p63 isoform (TAp63) and p53 resulting in apoptosis[19]. Source data are provided as a Source Data file.

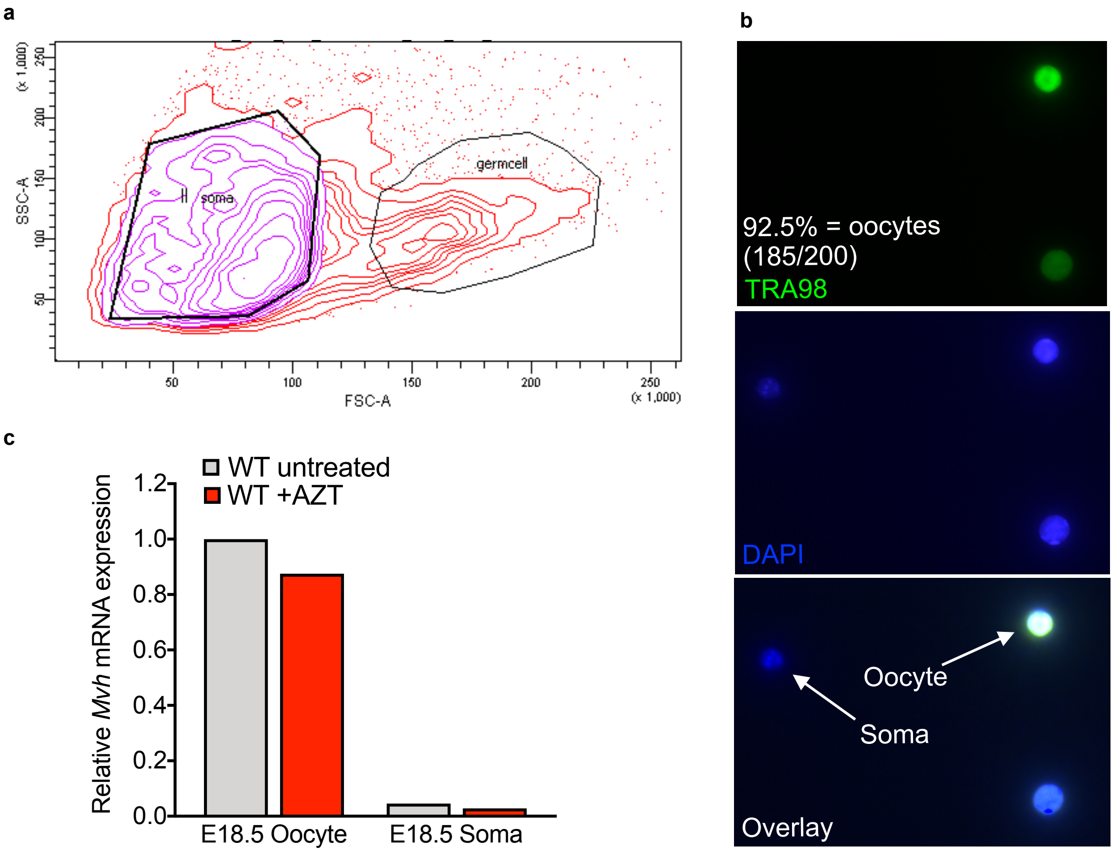

Supplementary Figure 5: Strategy to sort oocytes by FACS and oocyte purity assessments. (**a**) FACS profile during isolation of oocytes (germ cell) from ovarian somatic cells (soma) based on side and forward scatter parameters. Propidium iodide staining was also used to eliminate dead cells that were positive for propidium iodide fluorescence (data not shown). (**b**) Analysis of oocyte purity in a representative sorted oocyte sample based on TRA98 and DAPI staining. TRA98-positive cells were counted as oocytes and TRA98-negative cells were counted as soma. Percent oocyte purity noted on image. (**c**) Analysis of oocyte purity in untreated and AZT-treated sorted oocytes and soma using qRT-PCR detection of the germ cell-specific gene *Mvh* normalized to the untreated oocyte sample with *Actb* as an internal control. n≥6 WT CD1 embryos from one litter per sample. Each bar represents one biological replicate.

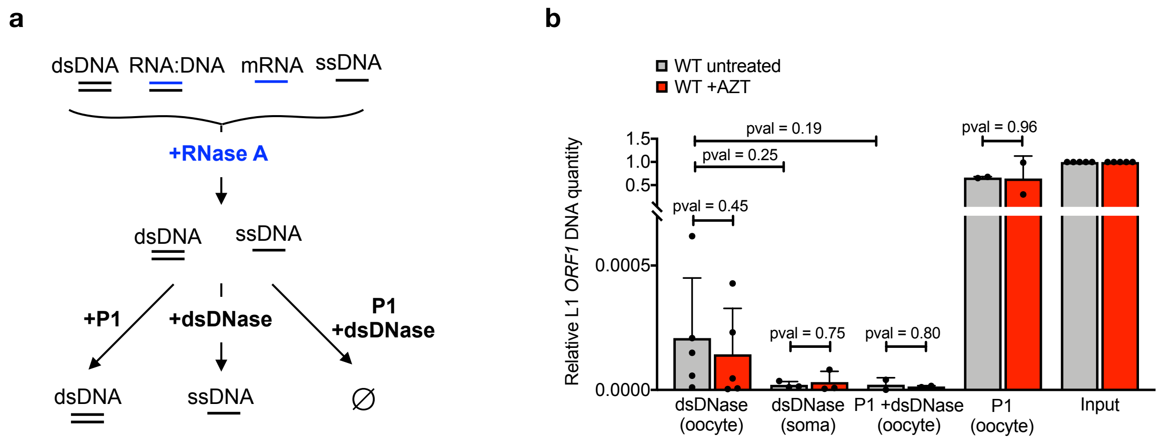

Supplementary Figure 6: Isolation and quantification of L1 single-stranded DNA. (a) Schematic for isolating L1 reverse transcription intermediates. Total DNA isolated from untreated and AZT-treated sorted oocytes and somatic cells after RNase A treatment to remove mRNA and RNA within RNA:DNA hybrids. $\geq$6 pairs of WT CD1 E16.5-E17.5 ovaries from single litter per sample. Subsequently, RNase A-treated DNA (input) was treated with double-stranded (ds) DNase to isolate single-stranded (ss) DNA (n=5 for oocytes and n=3 for somatic cells). Input DNA was also treated with the ssDNase P1 to control for dsDNA relative to ssDNA (n=2). Input DNA was also treated with P1 and dsDNase to control for residual dsDNA after dsDNase treatment (n=2). (**b)** Relative quantity of L1 *ORF1* DNA by quantitative PCR. Samples first normalized to the single copy gene *Ifnb1,* then to L1 *ORF1* of input DNA. Dots indicate individual biological replicates; data are mean +SD. Stats for comparison of untreated to AZT-treated samples by two-tailed paired Student's t-test, ns $p>0.05$; stats for comparison of WT untreated and AZT-treated, dsDNase-treated oocytes to WT untreated and AZT-treated negative controls by Mann-Whitney test, ns $p>0.05$. Source data are provided as a Source Data file.

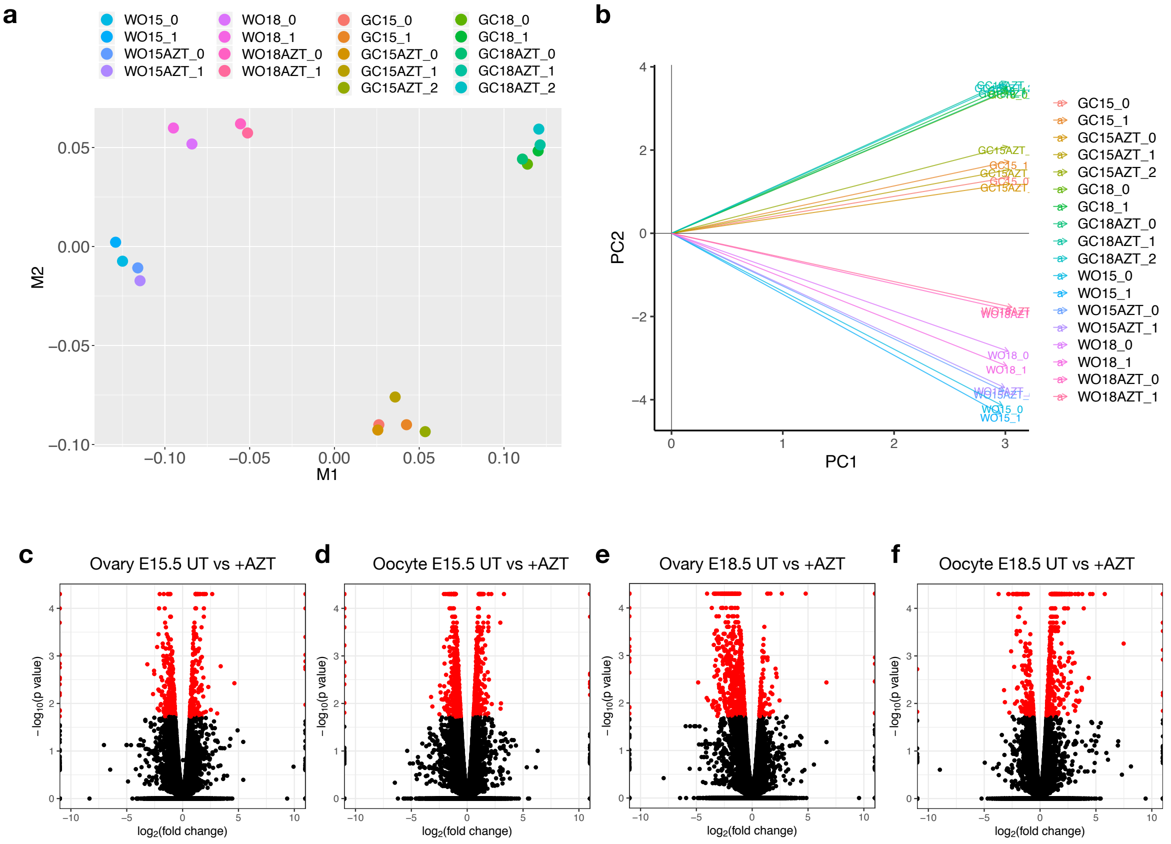

Supplementary Figure 7: Untreated and AZT-treated whole ovary and sorted oocyte mRNA-Seq. (a, b) Analysis of variation between E15.5 and E18.5, untreated at AZT-treated, whole ovary (WO) and sorted oocyte or germ cell (GC) samples using (**a**) Multidimensional scaling (MDS) and (**b**) Principle component analysis (PCA). Both MDS and PCA are dimensionality reduction techniques that determine variation between samples, however, PCA is better for inferring the contribution of specific genes to the separation of samples. Each sample contains CD1 ovaries or oocytes from at least one litter of female embryonic ovaries. E15.5 and E18.5 untreated and AZT-treated ovaries were performed in duplicate. E15.5 and E18.5 untreated oocytes were performed in duplicate. E15.5 and E18.5 AZT-treated oocytes were performed in triplicate. **(c-f)** Volcano plots displaying pairwise comparison of differential gene expression between (**c**) E15.5 untreated and E15.5 +AZT ovaries, (**d**) E15.5 untreated and E15.5 +AZT oocytes, (**e**) E18.5 untreated and E18.5 +AZT ovaries, and (**f**) E18.5 untreated and E18.5 +AZT oocytes (Supplementary Table 3a-d). Red dots indicate fold change difference is significant and black dots indicate not significant.

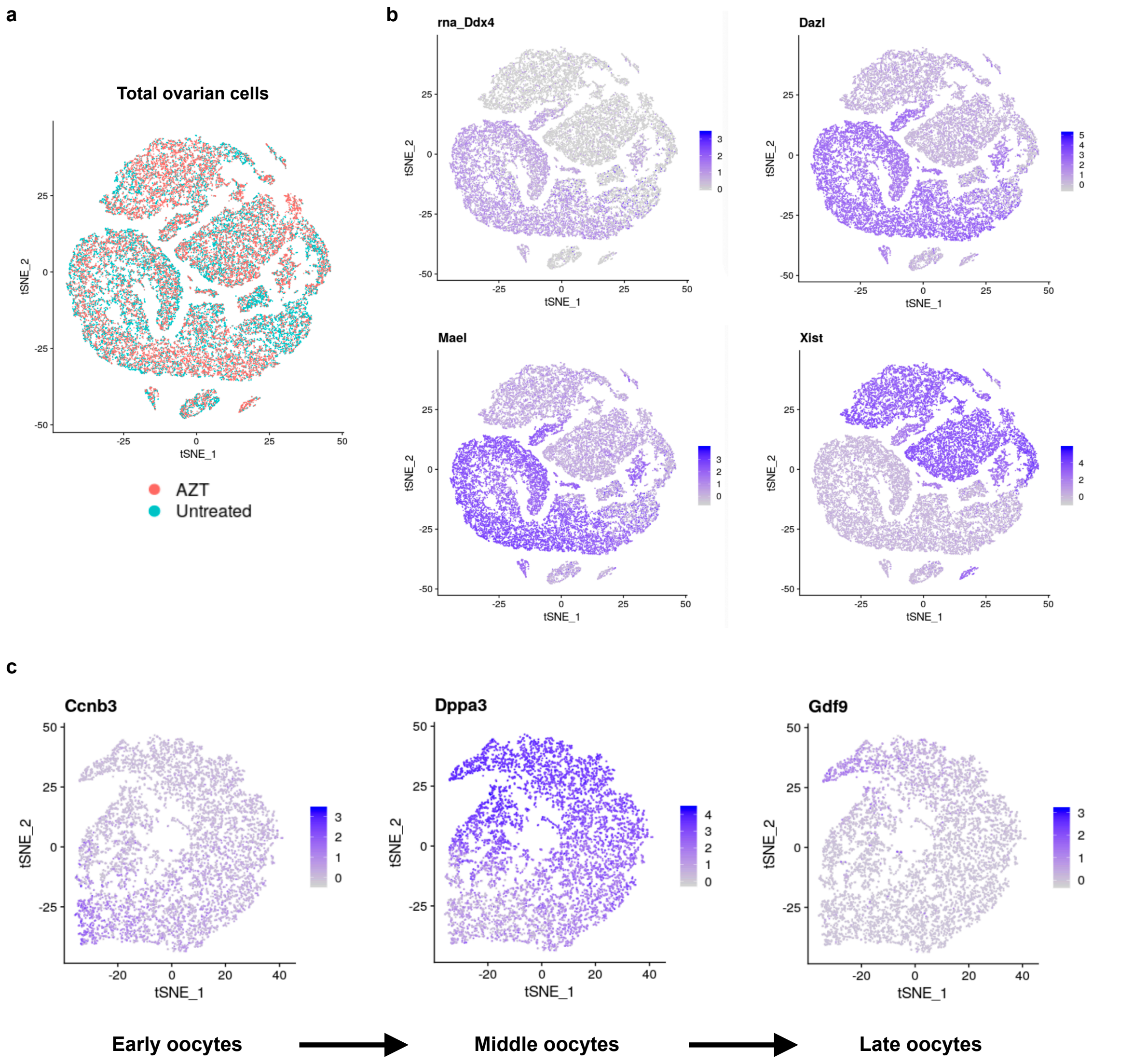

Supplementary Figure 8: Ovary single-cell transcriptome clustering and oocyte sub-setting. **(a)** T-SNE plot showing integration and overlay of E18.5 untreated and AZT-treated ovary transcriptomes. Untreated ovarian cells in blue and AZT-treated ovarian cells in orange. **(b)** T-SNE plot of integrated untreated and AZT-treated individual ovarian cells showing Ddx4, Dazl, Mael, and Xist expression used for sub-setting oocytes from somatic cells in Fig. 4a-d. Cells with average log fold change >0.5 for *Ddx4*, *Dazl*, and *Mael* and average log fold change expression <0.5 for *Xist* that is restricted to somatic cells were considered oocytes. (**c**) Expression of early, middle, and late oocyte markers in integrated E18.5 untreated and AZT-treated sub-set oocytes. *Ccnb3* is expressed in oocytes in early meiotic prophase I stages, *Dppa3* is expressed in most oocytes and is used as a marker of oocytes in the middle of the developmental trajectory, and *Gdf9* is expressed in late stage oocytes or primordial follicles. Ovaries from at least 6 CD1 embryos from a single litter were used per sample. Experiment was repeated one time each for untreated and AZT-treated samples, but with fewer cells sequenced (~5,000 cells per sample) and fewer reads/cell sequenced (~7,000-10,000 reads/cell) (replicate data not shown).
