# Supplementary material for: Maximizing the ovarian reserve in mice by evading LINE-1 genotoxicity
Source: Nat Commun. 2020 Jan 16;11:330. doi: 10.1038/s41467-019-14055-8 (PMC6965193; doi:10.1038/s41467-019-14055-8)
Supplement: Supplementary file 2 — Description of Additional Supplementary Files [file 41467_2019_14055_MOESM2_ESM.pdf]

Description of Additional Supplementary Files

File Name: Supplementary Data 1
Description: Quantification and statistical analysis for *Chk2*$^{+/-}$ and *Chk2*$^{-/-}$ untreated and AZT-treated ovary experiments.

File Name: Supplementary Data 2
Description: Quantification and statistical analysis for meiotic progression in *Chk2*$^{+/-}$ and *Chk2*$^{-/-}$ oocytes.

File Name: Supplementary Data 3
Description: Differential gene expression and gene ontology for untreated and AZT-treated oocytes and ovaries.

File Name: Supplementary Data 4
Description: Small RNA length distribution and repeat alignment.

File Name: Supplementary Data 5
Description: Quantification and statistical analysis for *Mili;Chk*2 untreated and AZT-treated ovary experiments.

File Name: Supplementary Data 6
Description: Differential gene expression in untreated and AZT-treated single-cell clusters.

File Name: Supplementary Data 7
Description: Quantification of follicles and fertility in *Chk2*$^{+/-}$ untreated and *Chk2*$^{-/-}$ untreated and AZT-treated mice.
