# Supplementary material for: Maximizing the ovarian reserve in mice by evading LINE-1 genotoxicity
Source: Nat Commun. 2020 Jan 16;11:330. doi: 10.1038/s41467-019-14055-8 (PMC6965193; doi:10.1038/s41467-019-14055-8)
Supplement: Supplementary file 10 — Reporting Summary [file 41467_2019_14055_MOESM10_ESM.pdf]

Corresponding author(s): Alex Bortvin
Last updated by author(s): Nov 18, 2019

# Reporting Summary

Nature Research wishes to improve the reproducibility of the work that we publish. This form provides structure for consistency and transparency in reporting. For further information on Nature Research policies, see Authors & Referees and the Editorial Policy Checklist.

## Statistics

For all statistical analyses, confirm that the following items are present in the figure legend, table legend, main text, or Methods section.

| n/a | Confirmed | |
|---|---|---|
| ☐ | ☒ | The exact sample size (*n*) for each experimental group/condition, given as a discrete number and unit of measurement |
| ☐ | ☒ | A statement on whether measurements were taken from distinct samples or whether the same sample was measured repeatedly |
| ☐ | ☒ | The statistical test(s) used AND whether they are one- or two-sided<br>*Only common tests should be described solely by name; describe more complex techniques in the Methods section.* |
| ☒ | ☐ | A description of all covariates tested |
| ☒ | ☐ | A description of any assumptions or corrections, such as tests of normality and adjustment for multiple comparisons |
| ☐ | ☒ | A full description of the statistical parameters including central tendency (e.g. means) or other basic estimates (e.g. regression coefficient) AND variation (e.g. standard deviation) or associated estimates of uncertainty (e.g. confidence intervals) |
| ☐ | ☒ | For null hypothesis testing, the test statistic (e.g. *F*, *t*, *r*) with confidence intervals, effect sizes, degrees of freedom and *P* value noted<br>*Give P values as exact values whenever suitable.* |
| ☒ | ☐ | For Bayesian analysis, information on the choice of priors and Markov chain Monte Carlo settings |
| ☒ | ☐ | For hierarchical and complex designs, identification of the appropriate level for tests and full reporting of outcomes |
| ☒ | ☐ | Estimates of effect sizes (e.g. Cohen's *d*, Pearson's *r*), indicating how they were calculated |

*Our web collection on statistics for biologists contains articles on many of the points above.*

## Software and code

Policy information about availability of computer code

| | |
|---|---|
| Data collection | Illumina sequencing data collection described in Methods section of Extended Data Materials.<br>RNA-seq: Illumina Next-seq 500<br>Image Acquisition: LAS AF<br>Flow Cytometry: BD FACS Aria III |
| Data analysis | Software packages used for data analysis described in Methods. Resulting differential gene expression and read length distribution results shown in Supplementary Tables 3, 4, and 6.<br>RNA-seq: TopHat aligner, Cuffdiff and cummeRbund package for differential gene expression analysis<br>Small RNA-seq: piPipes<br>Single-cell RNA-seq: CellRanger version 3.0.2, Seurat version 3.0<br>Image Analysis: Imaris (Bitplane) version 9.0<br>Flow Cytometry: BD FACSDiva<br>Statistical Analysis: Graphpad Prism Version 7.0 |

For manuscripts utilizing custom algorithms or software that are central to the research but not yet described in published literature, software must be made available to editors/reviewers. We strongly encourage code deposition in a community repository (e.g. GitHub). See the Nature Research guidelines for submitting code & software for further information.

## Data

Policy information about availability of data

All manuscripts must include a data availability statement. This statement should provide the following information, where applicable:

- Accession codes, unique identifiers, or web links for publicly available datasets
- A list of figures that have associated raw data
- A description of any restrictions on data availability

The NCBI Sequence Read Archive project number for all high-throughput sequencing data is: PRJNA543598. Fig. 2a-b, 3a, 3d, 3f-i, 4a-d and Supplementary Fig. 7a-f, 8a-c have associated raw data.

# Field-specific reporting

Please select the one below that is the best fit for your research. If you are not sure, read the appropriate sections before making your selection.

☒ Life sciences ☐ Behavioural & social sciences ☐ Ecological, evolutionary & environmental sciences

For a reference copy of the document with all sections, see nature.com/documents/nr-reporting-summary-flat.pdf

# Life sciences study design

All studies must disclose on these points even when the disclosure is negative.

| | |
|---|---|
| Sample size | Sample size was not predetermined, but followed accepted sample sizes from related studies. Sample sizes described in Supplementary Table 1, 2, 5, and 7. For each experimental condition, at least 3 ovaries from 3 different embryos and 2 different litters were used with few exceptions indicated in Supplementary Tables listed above. |
| Data exclusions | No data exclusions. |
| Replication | At least two biological replicates used for Illumina sequencing and qRT-PCR experiments, each replicate containing oocytes from at least three pairs of embryonic ovaries with few exceptions indicated in Methods or figure legends. Single cell RNA sequencing experiments were replicated once with smaller numbers of cells per sample (data not shown). |
| Randomization | No randomization. |
| Blinding | Blinding was used when selecting individual oocyte nuclei to determine L1 ORF1p or gamma H2AX fluorescence intensity. For other experiments, investigators were not blinded. |

# Reporting for specific materials, systems and methods

We require information from authors about some types of materials, experimental systems and methods used in many studies. Here, indicate whether each material, system or method listed is relevant to your study. If you are not sure if a list item applies to your research, read the appropriate section before selecting a response.

### Materials & experimental systems

| n/a | Involved in the study | |
|---|---|---|
| ☐ | ☒ | Antibodies |
| ☒ | ☐ | Eukaryotic cell lines |
| ☒ | ☐ | Palaeontology |
| ☐ | ☒ | Animals and other organisms |
| ☒ | ☐ | Human research participants |
| ☒ | ☐ | Clinical data |

### Methods

| n/a | Involved in the study | |
|---|---|---|
| ☒ | ☐ | ChIP-seq |
| ☐ | ☒ | Flow cytometry |
| ☒ | ☐ | MRI-based neuroimaging |

## Antibodies

| | |
|---|---|
| Antibodies used | Described in Antibodies section of Methods. |
| Validation | L1 ORF1p antibody validated using mouse Maelstrom-/- testis sections in: S. F. Soper et al., Mouse maelstrom, a component of nuage, is essential for spermatogenesis and transposon repression in meiosis. Dev Cell 15, 285-297 (2008). Other primary and secondary antibodies are widely used and validated commercial antibodies. |

## Animals and other organisms

Policy information about studies involving animals; ARRIVE guidelines recommended for reporting animal research

| | |
|---|---|
| Laboratory animals | Mouse strains CD1, C57Bl/6 and 129X1/Sv were used for experiments. Female embryos and pups were used with ages indicated in respective figures. |
| Wild animals | n/a |
| Field-collected samples | n/a |
| Ethics oversight | All experimental procedures were performed in compliance with ethical regulations and approved by the IACUC of the Carnegie Institution for Science. |

Note that full information on the approval of the study protocol must also be provided in the manuscript.

## Flow Cytometry

### Plots

Confirm that:

☒ The axis labels state the marker and fluorochrome used (e.g. CD4-FITC).

☒ The axis scales are clearly visible. Include numbers along axes only for bottom left plot of group (a 'group' is an analysis of identical markers).

☒ All plots are contour plots with outliers or pseudocolor plots.

☒ A numerical value for number of cells or percentage (with statistics) is provided.

### Methodology

| | |
|---|---|
| Sample preparation | Described in FACS sorting section of Methods. |
| Instrument | BD FACS Aria III |
| Software | BD FACSDiva software |
| Cell population abundance | Purity of samples determined by immunofluorescence and qRT-PCR shown in Supplementary Fig. 5b-c. |
| Gating strategy | Described in FACS sorting section of Methods in Extended Data Materials and Supplementary Fig. 5a. |

☒ Tick this box to confirm that a figure exemplifying the gating strategy is provided in the Supplementary Information.
